# *MSTN* knockout enhances the production of *MYOD1*-mediated steak-type cultivated meat

**DOI:** 10.1186/s40104-025-01173-1

**Published:** 2025-03-11

**Authors:** Kyeong-Hyeon Eom, Dayi Jeong, Jae-Yoon Choi, Gyeong-Min Gim, Soo-Young Yum, Sumin Jin, Hojae Bae, Goo Jang

**Affiliations:** 1 Department of Theriogenology and Biotechnology, College of Veterinary Medicine and the Research Institute for Veterinary Science, Seoul National University, 1 Gwanak-Ro, Gwanak-Gu, Seoul, 08826 Republic of Korea; 2 LART Bio Inc, 60 Haan-Ro, Gwangmyeong-Si, Gyeonggi-Do, 14322 Republic of Korea; 3 Department of Stem Cell and Regenerative Biotechnology, KU Convergence Science and Technology Institute, Konkuk University, Seoul, 05029 Republic of Korea; 4 Institute of Advanced Regenerative Science, Konkuk University, Seoul, 05029 Republic of Korea; 5 Comparative Medicine Disease Research Center, Seoul National University, 1 Gwanak-Ro, Gwanak-Gu, Seoul, 08826 Republic of Korea

**Keywords:** Cultivated meat, Digital light processing, Gelatin methacryloyl (GelMA) hydrogels

## Abstract

**Background:**

As the global population increases, the demand for protein sources is expected to increase, driving the demand for cell-based cultivated meat. This study aimed to enhance the productivity of cultivated meat through optimization of the cell source and organization process.

**Results:**

We engineered fibroblasts into myogenic cells via non-viral introduction of the *MYOD1* gene, avoiding viral methods for safety. After confirming the stable derivation of myogenic cells, we combined knockout (KO) of *MSTN*, a negative regulator of myogenesis, with *MYOD1*-mediated myogenesis to improve cultivated meat production. Primary cells from *MSTN* KO cattle exhibited enhanced myogenic potential. Additionally, when tested in immortalized fibroblasts, myostatin treatment reduced *MYOD1*-induced myogenesis in two-dimensional cultures, while *MSTN* knockout increased it. To achieve muscle-like cell alignment, we employed digital light processing (DLP)-based three-dimensional (3D) bioprinting to organize cells into 3D groove-shaped hydrogels. These bioactive hydrogels supported stable cell proliferation and significantly improved muscle cell alignment. Upon differentiation into myotubes, the cells demonstrated an ordered alignment, particularly the *MSTN* KO cells, which showed highly efficient differentiation.

**Conclusions:**

The integration of genetic modification and advanced DLP 3D bioprinting with groove-patterned hydrogels provides an effective strategy for producing high-quality, muscle-aligned cultivated meat.

**Supplementary Information:**

The online version contains supplementary material available at 10.1186/s40104-025-01173-1.

## Background

As the global population continues to increase, the demand for protein sources is also expected to increase, potentially leading to a global food crisis [1, 2]. This issue is particularly significant for beef. Although beef has the highest feed conversion ratio among the major livestock products, global beef consumption is predicted to grow over the next ten years, driving the demand for alternative sources such as cultivated meat [3–6]. In this study, our objective was to improve the productivity of cultivated beef by optimizing the cell source and improving the organizational process.

Different cell types have been used for cultivated meat production. Primary myoblasts or satellite cells isolated from muscle tissues are primarily used because of their biological similarities with conventional meat. Myoblasts can be obtained from slaughtered animals or, ideally, through tissue biopsy. However, the biopsy procedure not only requires chemical and/or physical restraints but is also limited in utility, as the proportion of satellite cells, which can constitute up to 30% of muscle nuclei in neonates, decreases rapidly with aging [5, 7]. Moreover, isolated primary myoblasts exhibit limited doubling (~ 50 doublings), which is a significant challenge [5]. Pluripotent stem cells have been suggested to address the proliferation problem; however, culturing pluripotent stem cells requires highly skilled handling. The difficulties in obtaining embryos, and in proliferating and maintaining these stem cells are barriers that make this option unsuitable for most researchers [8, 9]. To address the limitations of other options, the conversion of fibroblasts to myogenic cells by overexpressing MyoD has been adopted as an alternative [10–13]. Fibroblasts not only constitute the largest proportion of cells in the body but are also highly susceptible to MYOD-induced trans-differentiation due to their mesodermal origin, similar to muscle cells [11, 14]. In cattle, fibroblasts can be sampled through a process similar to routine ear tagging and have been stably cultured from adult animals [15, 16]. Furthermore, fibroblasts are among the most extensively studied cell types, with a variety of immortalization strategies having been reported [17–19]. In a previous study, we produced centimeter-scale cultivated meat using myogenic cells that have been converted from non-myogenic cells [11, 20].

Regarding the cell source, our objective was to develop a cell line that is efficient and safe for consumers when used for the production of cultivated meat. First, we verified *MYOD1*-mediated conversion using a non-viral transfection system in primary and immortalized fibroblasts, which were also produced by introducing non-viral telomerase reverse transcriptase (TERT) [17]. We then verified whether this myogenic conversion was maintained in immortalized TERT-induced cells to provide a stable and myogenic cell line for the production of cultivated meat.

To improve conversion efficiency, we targeted the endogenous *MSTN* gene using the CRISPR/Cas9 system. Myostatin, encoded by the *MSTN* gene, is a well-conserved negative regulator of myogenesis across species [21, 22]. Naturally occurring mutations or variants in the *MSTN* gene have been identified in domestic animals, including cattle, pigs, horses, sheep, and dogs, and these mutations are associated with the muscular hypertrophy or “double-muscling” phenotype [21, 23, 24]. Based on these findings, genome-edited animals with *MSTN* mutations have been produced across various species to enhance productivity [25–29]. Myostatin is known to bind activin receptor type IIA and IIB receptors, activating Smad3 by inducing its phosphorylation. The phosphorylated Smad3 forms a complex with Smad4, moves into the nucleus, and regulates the expression of myogenesis-related transcription factors [30, 31]. Myostatin inhibits myogenesis primarily by downregulating the expression of the MyoD during natural myoblast differentiation [30]. However, its effect on *MYOD1*-induced conversion cells remains unclear. To investigate this, we analyzed myogenic conversion efficiency in MSTN-mutated bovine primary cells. Additionally, we examined the role of myostatin in *MYOD1*-induced myogenesis by treating immortalized cell lines with myostatin or knocking out the *MSTN* gene.

Building on these advances in cell line development, we used digital light processing (DLP) three-dimensional (3D) bioprinting, a photopolymerization-based technology that utilizes digital mask projection to fabricate a structural framework for cultivated meat. DLP 3D bioprinting technology enables the enlargement of scaffolds containing internalized cells with high precision and speed [32, 33]. In a previous study, we demonstrated the feasibility of producing centimeter-sized steak-like cultivated meat using DLP 3D printing technology [34]. Precision-controlled microchannels within the scaffold facilitate the delivery of nutrients to cells, even at centimeter-scale thicknesses.

Numerous studies have aimed to simulate muscle cell alignment in 3D structures [35]. Methods such as applying electrical signals to hydrogels [36], providing physical stimulation to hydrogels [37], or producing thin fiber strands [38] are effective for muscle alignment. However, a faster and more streamlined method is required to enhance the economics and productivity of cultivated meat [39]. While DLP 3D bioprinting technology shows significant potential for rapid production, further investigation is required to optimize its application for achieving effective muscle cell alignment.

*MSTN* knock-out (KO) cells were encapsulated within gelatin methacryloyl (GelMA) hydrogel bioink and multichannel-grooved steak-like scaffolds were fabricated using DLP 3D printing. The high biocompatibility and cell viability properties of GelMA make it suitable as a cell encapsulation bioink [40]. Several studies have reported on the fabrication of micropatterned substrates with microgroove structures to promote myoblast alignment [41, 42]. However, hydrogels with microgrooves are typically based on two-dimensional platforms, and these approaches lack scalability, limiting their application in the development of cultivated meat. To overcome these scalability limitations, we printed 100-μm-scale groove patterned structures in addition to hydrogels containing microchannels to promote muscle cell alignment and induce muscle cells to proliferate and differentiate along the groove structures.

In this study, we aimed to advance the field of cultivated meat production by integrating *MSTN* KO cells with advanced DLP 3D bioprinting techniques. This approach is a scalable and efficient method to generate high-quality muscle-aligned cultivated meat that closely replicates the properties of conventional meat. This innovative approach has the potential to address the increasing global demand for sustainable protein sources while addressing critical challenges in food safety and production efficiency.

## Methods

Tartrazine was purchased from GreenTech (Daejeon, South Korea). The Live/Dead cell viability kit was purchased from Invitrogen (Carlsbad, CA, USA). Dulbecco’s modified Eagle medium (DMEM), penicillin streptomycin (p/s), and fetal bovine serum (FBS), were all from Gibco™, Thermo Fisher Scientific Inc. (Waltham, MA, USA). Saline buffered with L-glutamine and phosphate (PBS, pH 7.4) was purchased from WelGene (Daegu, Gyeongbuk, South Korea). Gelatin (Type A, 300 bloom from porcine skin), methacrylic anhydride (MA), lithium phenyl-2,4,6-trimethylbenzoylphosphinate (LAP, ≥ 95%), doxycycline, insulin, bovine serum albumin and Triton X-100 were purchased from Sigma-Aldrich (St. Louis, MO, USA). All other chemicals used in this study were analytical grade.

### *MYOD1* gene identification and vector cloning

The bovine *MYOD1* coding sequences were identified in the cattle genome (ARS-UCD 1.3) and synthesized using a commercial gene synthesis service (VectorBuilder, Chicago, IL, USA). The synthesized gene was cloned into the Piggybac transposon vector along with promoters or reporter genes as needed in the experiments using overlapping PCR-based cloning kits (Takara Bio, Inc., Kusatsu, Japan).

### Cell culture

Primary cells were obtained via biopsy of calf ear skin. The skin samples were cut into small pieces with a sterile scalpel, then washed several times, and incubated at 38.5 °C for 4–18 h in Hank’s Balanced Salt Solution (Thermo Fisher Scientific Inc.), supplemented with type I collagenase (Thermo Fisher Scientific Inc.). The following day, the dispersed cells were washed several times in DMEM and cultured in growth medium (GM; DMEM with 20% FBS and 1% p/s). For *MSTN* mutant cells, primary cells were derived from the ear skin of *MSTN* mutant cattle as previously reported [25, 26] and cultured at 38 °C and in a 5% CO_2_ humidified atmosphere. For (wildtype) WTcells, primary cells at the same age as their knockout counterparts were derived from the ear skin. A bovine immortalized fibroblast cell line established in a previous study was used in this study [17]. The immortalized fibroblasts were cultured under identical conditions. When passaging the cells, they were detached enzymatically using TrypLE dissociation reagents (Thermo Fisher Scientific Inc.). The detached cells were stained with Trypan blue stain and counted using an automatic cell counter following the manufacturer’s instructions (Thermo Fisher Scientific Inc.).

### Cell transfection, mutation analysis, and tetracycline treatment

Depending on the purpose of each experiment, vectors or the Cas9-gRNA complex (RNP) were introduced into cells using a NEON™ transfection kit (Invitrogen), following the manufacturer’s instructions. The electroporation conditions were 1,400 V/20 ms/2 pulses. The RNP was composed of 100 ng/μL Cas9 protein and 200 ng/μL of guide RNA. For transposon vectors, 500 ng each of the vectors of interest and vectors encoding transposase were co-transfected. Genomic DNA was extracted from the cells using a DNA extraction kit (Qiagen, 69504) and the extracted DNA was used for PCR and Sanger sequencing. The sequencing results were analyzed using Synthego ICE software (https://ice.editco.bio). The *MSTN* gene mutation detection primers were as follows: forward: 5′-GAGGTGTTCGTTCGTTTTTC-3′, reverse: 5′-TAAGCACAGGAAACTGGTAG-3′. Unless indicated otherwise, for the activation of the conditional Tet-on promoter, 2 μg/mL doxycycline (Sigma-Aldrich) was added to GM (MDM; myogenic differentiation medium).

### Immunocytochemistry and fusion index

Cells were seeded in 6-well plates and overexpression of the *MYOD1* gene was induced by doxycycline treatment for 7 d. After appropriate culture periods, cells were fixed with 4% paraformaldehyde (Biosesang, Yong-in, Gyeong-gi, South Korea) for 10 min at room temperature and permeabilized with 1% Triton X-100 in PBS for 10 min also at room temperature. The fixed cells were washed at least three times with PBS and blocked with 1% bovine serum albumin (Gendepot, Katy, TX, USA) in PBS. After the blocking step, cells were incubated with primary antibody overnight at 4 °C. The primary antibodies included anti-desmin antibody (Sigma-Aldrich), anti-MYH1/2 antibody (Santa Cruz Biotech, Dallas, TX, USA), and anti-α-actinin (sarcomeric) antibody (Sigma-Aldrich). The actin filaments were labelled with Alexa Fluor™ 555 phalloidin (Invitrogen). On the following day, cells were washed at least five times and incubated with DyLight™ 488 conjugated anti-mouse IgG antibody (Invitrogen), Alexa Fluor™ 488 conjugated anti-rabbit IgG antibody (Invitrogen), and Alexa Fluor™ 594 conjugated anti-mouse IgG antibody (Invitrogen) for 1 h. After at least five rinses with PBS, the nuclei were stained with 1 μg/mL of Hoechst 33342 stain solved in PBS. After the last wash, cells were observed using an EVOS m5000 or m7000 (Thermo Fisher Scientific Inc.). 3D cell imaging was performed using a Lionheart FX automated microscope (BioTek, Winooski, VT, USA) or Zeiss laser scanning confocal microscopy (LSM700, Zeiss, Germany). Z-stack images were obtained using the Z-projection feature in Gen5 Image Prime 3.09 software (BioTek, Winooski, VT, USA). For fusion index calculation, Hoechst images and immunofluorescence images stained for desmin were merged and the number of nuclei within same cell membrane was counted using ImageJ software and divided by the total number of nuclei. At least three random images of each test group were used for the fusion index calculations. For cells with green and red fluorescence, a pre-conjugated anti-desmin antibody (Abcam, Cambridge, UK) was used for immunocytochemistry (ICC) and monitored in m7000 using a Texas Red color (TxRed) laser.

### Quantitative real-time polymerase chain reaction

Total RNA was extracted from cells treated with vectors or RNPs using an RNeasy Mini Kit (Qiagen, Hilden, Germany) to measure expression levels Complementary deoxyribonucleic acid (cDNA) was synthesized from 1–2 μg of RNA using the RNA to cDNA EcoDry™ Premix (Takara Bio, Inc.). Gene expression assay was conducted using SYBR™ Green (Thermo Fisher Scientific Inc.) on a QuantStudio™ 3 real-time polymerase chain reaction (PCR) system (Thermo Fisher Scientific Inc.), and the relative cycle threshold (Ct) values were normalized to glyceraldehyde 3-phosphate dehydrogenase (GAPDH). For the immortalized fibroblasts, delta-delta-Ct values were used for analysis. For the primary cells, delta-Ct values were used for analysis, considering the individual differences in gene expression. At least three values for each technical repeat were included in the analysis. The primer sequences for the target genes were as follows: *GAPDH* forward: 5′-GGGTCATCATCTCTGCACCT-3′, reverse: 5′-GGTCATAAGTCCCTCCACGA-3′; *MYOD1* forward: 5′-GTGCAAACGCAAGACGACTA-3′, reverse: 5′-GCTGGTTTGGGTTGCTAGAC-3′; *MYOG* forward: 5′-TGGGCGTGTAAGGTGTGTAA-3′, reverse: 5′-TGCAGGCGCTCTATGTACTG-3′; *MYF5* forward: 5′-AGACGCCTGAAGAAGGTCAA-3′, reverse: 5′-AGCAGCTCCTGCAGACTCTC-3′; *MYF6* forward: 5′-TTACCCTGCAGCCCTTAGAA-3′, reverse: 5′-AAGCCCAGATCAGACATTGG-3′; *DESMIN* forward: 5′-GGGACATCCGTGCTCAGTAT-3′, reverse: 5′-GTGGCGGTACTCCATCATCT-3′; Myosin heavy chain 1 (*MYH1*) forward: 5′-GGCCAGACTGTAGAGCAGGTAT-3′, reverse: 5′-GGCAACCATCCACAGGAACATC-3′; *ACTN2* forward: 5′-AGAACGAGGTGGAGAAGGTG-3′, reverse: 5′-GCTTCACCTTGTCCCACTTG-3′. The thermal cycling conditions were: 95 °C for 10 min, followed by 40 cycles at 95 °C for 15 s, and 60 °C for 1 min.

### Tumorigenicity assay with immunodeficient mice

All experimental procedures were approved by the Institutional Animal Care and Use Committee of the Institute of Laboratory Animal Resources, Seoul National University (Approval No. SNU-230906-2-2). Three-week-old female BALB/c *nu*/*nu* mice were purchased and acclimated for 14 d before treatment (Raon Bio, Yong-in, Gyeong-gi, South Korea). Animals were kept in a pathogen-free environment with a controlled 12-h light/12-h dark cycle and had ad libitum access to a standard rodent diet and water. Cells for injection were trypsinized and suspended in PBS. Live cells were counted using a Countess II (Invitrogen) and diluted to a concentration of 100,000 cells/100 µL of PBS. Prepared cell suspensions were injected subcutaneously into the intrascapular region using 1 mL syringes with a 23 G needle into 5-week-old mice (*n* = 3 or 5). The mice were palpated at each timepoint for 22 d to observe nodule formation at the injection site. Body weight and tumor size were also measured, and a picture of each mouse was taken. Tumor size was assessed by external measurement of the length and width of the tumors in two dimensions. The tumor volume was calculated using the formula: volume = 0.5 × length (mm) × width^2^ (mm^2^). Mice were euthanized when tumors reached approximately 20 mm in any dimension or when signs of deconditioning were noted. All animals were sacrificed under CO_2_ inhalation anesthesia, and all efforts were made to minimize suffering.

### Western blotting

For western blotting, the harvested cells were lysed using RIPA lysis buffer (Thermo Fisher Scientific Inc.) supplemented with a proteinase inhibitor (Thermo Fisher Scientific Inc.) on ice for 30 min. The lysed cells were centrifuged for 13,000 r/min at 4 °C. The supernatant was harvested and used to quantify protein concentration using protein broad-range assay kits following the manufacturer’s protocol (Thermo Fisher Scientific Inc.). For each group, 20 μg of protein was mixed with SDS-PAGE sample buffer (Biopeace, Dajeon, Chungnam, South Korea) and loaded into mini-protean TGX gels (Bio-Rad, Hercules, CA, USA). For the western blotting assay, the same volume of each medium was used without a quantification step. The loaded gel was run at 100 V for 80 min, and the proteins were transferred to a PVDF membrane (Bio-Rad) at 0.3A for 2 h. The transferred membrane was blocked with an appropriate blocking buffer (Biopeace) and incubated with an anti-myostatin antibody (R&D Systems, Minneapolis, MN, USA) or anti-pSmad3 antibody (Thermo Fisher Scientific Inc.) overnight at 4 °C. The next day, the membranes were washed and incubated with HRP peroxidase-conjugated secondary antibody (Gendepot) for 1 h at room temperature. Chemiluminescent bands were visualized using iBright 1500 (Thermo Fisher Scientific Inc.) after treatment with an enhancer-peroxide solution mixture (Biopeace).

### GelMA synthesis

GelMA hydrogel was synthesized according to a previously established protocol [34, 43]. Briefly, 10% (w/v) type A porcine skin gelatin was dissolved in PBS while stirring at 50 °C until fully dissolved. MA was added at a rate of 1 mL/min until a final concentration of 15% (v/v) was obtained. The mixture was allowed to react at 50 °C for 4 h. The reaction was then stopped by adding PBS at 40 °C at a volume ratio of 4:1. Subsequently, the mixture was dialyzed against distilled water for 1 week using dialysis tubing with a 12–14 kDa molecular weight cutoff to remove any unreacted MA. Finally, the dialyzed solution was lyophilized for 4 d to yield completely dry pure GelMA.

### Bioink preparation

To prepare the bioink for DLP bioprinting, lyophilized GelMA hydrogel was fully dissolved at 10% (w/v) in Dulbecco's PBS (DPBS) at 37 °C. The solution contained 1% (v/v) p/s, 2% (v/v) FBS, 0.5% (w/v) LAP, and 1% (v/v) tartrazine. The cultured negative control (NC) and *MSTN* KO cells were harvested from the culture plates and incorporated into GelMA solution at a concentration of 2 × 10^6^ cells/mL.

### Photorheological Analysis

Photorheological measurements were performed following our previous methods [34]. Using a HAKKE MARS 40 rheometer (Thermo Fisher Scientific Inc.) equipped with an accessory light module, we investigated the rheological behavior of the bioink under light-exposure conditions similar to those of the DLP printer. An OmniCure LX 505 (Lumen Dynamics; Mississauga, ON, Canada) with a 405-nm LED channel was used to generate the light source. The light intensity was maintained at 2.45 mW/cm^2^ and the irradiation was directed through a collimator and reflected onto the measuring glass. The LED intensity of the glass rheometer was calibrated to match the intensity of the DLP printer. About 100 μL of bioink was loaded between the glass and the bottom plate at intervals of 100 μm. An oscillatory shear strain of 0.008% was applied at 20 Hz.

### 3D modeling and printing

A muscle-mimicking steak-type cultivated meat structure was designed using a Fusion 360 platform (Autodesk, San Rafael, CA, USA). The design consisted of 11 repeating layers, each comprising fibers with a thickness of 700 μm, connected by bridges with a thickness of 900 μm. The microchannels were configured with specific dimensions depending on their location within the structure: the top part featured channels with a width of 900 μm and a height of 1 mm, the front part had channels with a width of 900 μm and a height of 500 μm, and the side part had channels with a width of 1 mm and a height of 900 μm. In the groove-shaped hydrogel (GSH), a uniform groove pattern was applied across both fibers and bridges, with each groove measuring 100 μm wide and 100 μm high. The designed structure had an overall width of 29 mm, one was 55.91 mm in length, and 9.6 mm in height. The stereolithography model was cut in 100-μm increments.

To fabricate muscle-mimicking steak-type meat, a 3D hydrogel construct loaded with *MSTN* KO or NC cells was printed on an IM2 DLP 3D printer (Carima, Seoul, Korea). The bioink reservoir was maintained at a temperature of 37 °C and filled with 20 mL of bioink. The optimal light exposure time was then determined and set accordingly. UV–visible light in the range of 365–420 nm was used with an intensity of 1.97 mW/cm^2^. The printing process involved the sequential layering of 96 layers, each with a focal thickness of 100 μm. Upon completion of printing, the steak-type hydrogel construct was carefully removed from the build plate, rinsed with DPBS containing 1% p/s (v/v), and subsequently incubated in cell culture medium.

### Cell proliferation and differentiation in hydrogel

The hydrogel constructs harboring NC or *MSTN* KO cells at a density of 2 × 10^6^ cells/mL were maintained in a humidified atmosphere at 38 °C with 5% CO_2_ in GM. To induce myogenic differentiation, MDM was added and the constructs were cultured for 7 d. The differentiation medium was refreshed every 2 d to ensure optimal conditions for differentiation. To measure cell numbers, cell-laden hydrogels were digested with 1 mg/mL collagenase-A solution for 20 min at 37 °C, and the retrieved cells were counted with a hemocytometer.

### Live/dead assay

To investigate the cytotoxicity of GelMA, the viability of 3D-printed cells was evaluated using a Live/Dead cell viability assay with calcein AM and ethidium homodimer-1. Cells were encapsulated in GelMA hydrogel at a density of 2 × 10^6^ cells/mL and cultured in a humidified atmosphere at 38 °C with 5% CO_2_. After incubation periods of 1, 3, 6, and 9 d, cells were transferred to 24-well plates and 500 μL of staining solution was added to each well following the manufacturer’s instructions. Cell imaging was performed using a Lionheart FX automated microscope (BioTek). Cell viability was determined by calculating the percentage of live cells relative to the total cell count.

### EdU assay

Cell proliferation within the 3D-printed hydrogel was evaluated using the Click-iT EdU Imaging Kit (Invitrogen). Briefly, cells encapsulated in the hydrogel were cultured under standard conditions and then incubated with 5-ethynyl-2′-deoxyuridine (EdU) according to the manufacturer’s protocol. Following incubation, the hydrogels were fixed in 4% paraformaldehyde for 15 min at room temperature and permeabilized using 0.5% Triton X-100 in PBS for 20 min. The EdU labeling reaction was performed by adding the Click-iT reaction cocktail to the hydrogels and incubating for 30 min, protected from light. After labeling, the samples were washed thoroughly with PBS and imaged using a Lionheart FX automated microscope (BioTek). Z-stack images were obtained using the Z-projection feature in the Gen5 Image Prime 3.09 software (BioTek) to visualize cell proliferation within both the surface and interior regions of the hydrogel.

### Mechanical properties

The compressive strength of steak-type cultivated meat and beef (tenderloin) was evaluated using a CT3 Texture Analyzer (Brookfield, Toronto, ON, Canada) equipped with a 4,500 g load cell in compression mode. Samples were prepared in two conditions (raw and pan-fried) to compare their mechanical properties. Cylindrical specimens with a diameter of 8 mm and a height of 8 mm were obtained using an 8 mm biopsy punch. A 12.7-mm diameter compression probe was used, applying a trigger load of 0.05 N at a testing speed of 0.05 mm/s. The compressive modulus was calculated as the slope of the linear region corresponding to 5%–15% strain, as defined by standard protocols [44].

### Statistical analysis

**Fig. 1:**
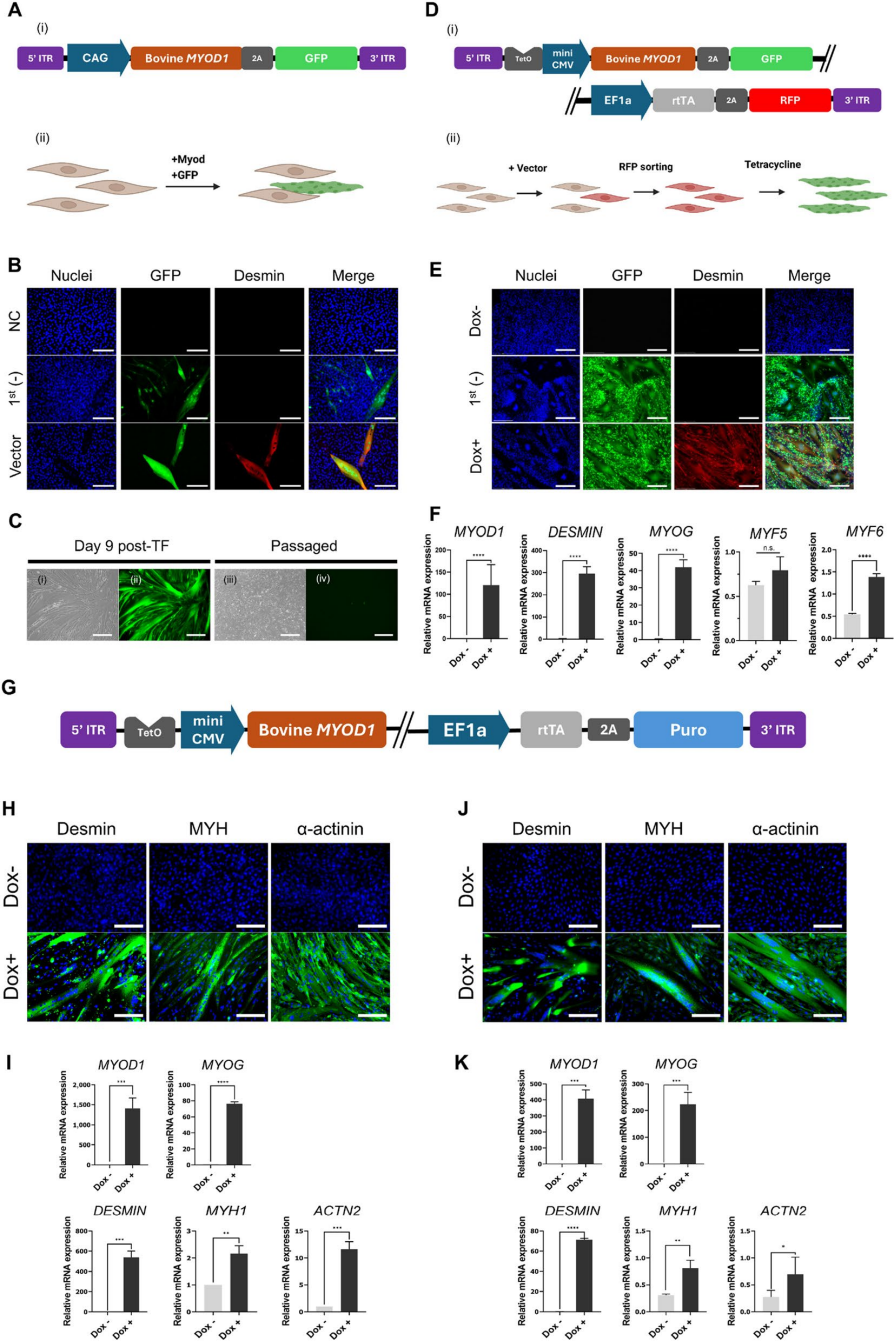
*MYOD1*-induced myogenic conversion of bovine fibroblasts using piggybac transposon. **A **Structure of *MYOD1*-expression vector with ubiquitous promoter (i) and experimental scheme (ii). **B** Immunofluorescence images using anti-desmin antibody in cells transfected with ubiquitous *MYOD1* expression. NC = Un-transfected negative control, 1^st^ (-) = Transfected vector, but stained without the primary antibody, Vector = Transfected with *MYOD1* vector. Green = GFP, Red = Desmin, and Blue = Nuclei. Scale bar = 150 μm. **C** Bright and fluorescent images of GFP reporter expression on day 9 after transfection before (i and ii) and after passaging (iii and iv). Scale bar = 275 μm. **D** Structure of conditional *MYOD1* expression vector (i) and experimental scheme (ii). 5′ ITR to 3′ ITR sequences were all packaged in single vector. **E** Immunofluorescence images using anti-desmin antibody. Cells integrated with vectors were sorted using RFP reporter and divided into following three groups. Dox- = untreated with Dox and stained using all antibodies, 1^st^ (-) = treated with Dox and stained without using the primary antibody, Dox+ = treated with Dox and stained using all antibodies. Green = GFP, TxRed = Desmin, and Blue = Nuclei. Scale bar = 275 μm. **F** Semi-quantitative real time PCR analysis of *MYOD1* and other myogenesis related factors, related to homeobox gene. Cells transfected with piggybac vectors were sorted using reporter, and 2 μg/mL of doxycycline was treated for 7 d before analysis. Data are presented as mean ± SD (n = 3). Asterisks indicate statistically significant differences (^****^P < 0.0001; n.s., not significant). **G** Structure of the dox-dependent *MYOD1* expression vector with puromycin resistance gene (**H**) immunofluorescence images of trans-differentiated primary bovine fibroblasts using *MYOD1*-puro vector. Dox- = untreated with doxycycline; Dox+ = treated with doxycycline. Green = immunofluorescent stain, Blue = Nuclei. Scale bar = 150 μm. **I** Semi-quantitative real time PCR analysis of *MYOD1* and other myogenesis markers, related to homeobox gene in trans-differentiated primary cells. Data are presented as mean ± SD (*n* = 3). Asterisks indicate statistically significant differences (^**^P < 0.01; ^***^P < 0.001; ^****^P < 0.0001; n.s., not significant). **J** immunofluorescence images of trans-differentiated bovine immortalized fibroblasts using *MYOD1*-puro vector. Dox- = untreated with doxycycline; Dox+ = treated with doxycycline. Green = immunofluorescent stain, Blue = Nuclei. Scale bar = 150 μm. **K** Semi-quantitative real time PCR analysis of *MYOD1* and other myogenesis markers, related to homeobox gene in trans-differentiated bovine immortalized cells. Data are presented as mean ± SD (*n* = 3). Asterisks indicate statistically significant differences (^*^P < 0.05; ^**^P < 0.01; ^***^P < 0.001; ^****^P < 0.0001; n.s., not significant)

**Fig. 2:**
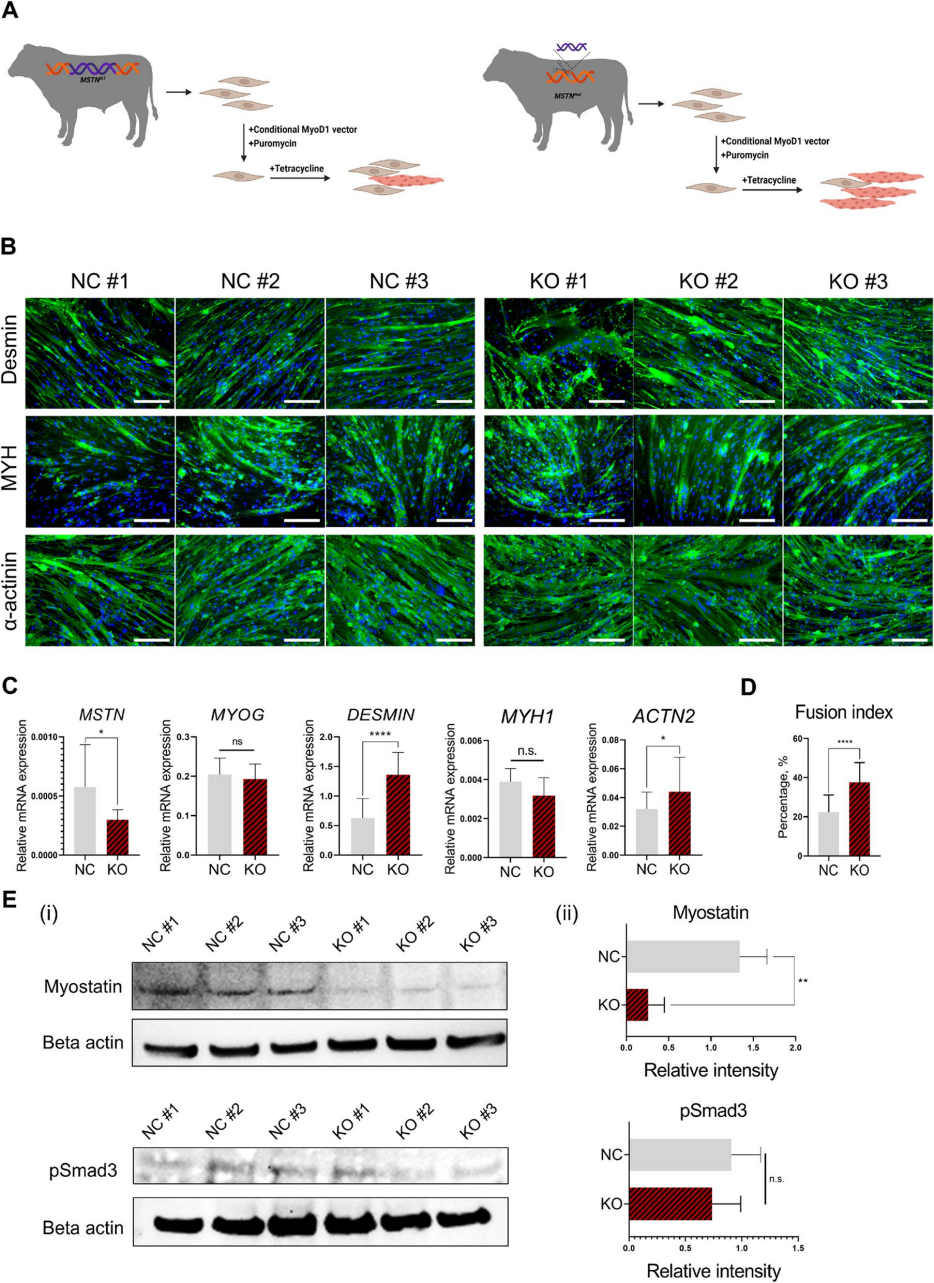
*MYOD1*-induced myogenic conversion of bovine fibroblasts from WT and *MSTN* KO cattle. **A** Scheme of experiments for Fig. 2B and C. **B** Immunofluorescence images of trans-differentiated primary bovine fibroblasts from WT or *MSTN* KO cattle. Green = immunofluorescent stain, Blue = Nuclei. Scale bar = 150 μm. **C** and **D** Semi-quantitative analysis of myogenesis factors expression (**C**) and fusion indexes (**D**) after myogenic conversion in fibroblasts from WT or *MSTN* KO cattle. The fibroblasts were derived from 3 different WT cattle (WT #1–#3) and 3 different *MSTN* KO cattle (KO #1–#3). 2 μg/mL of doxycycline was treated for 7 d for conditional myogenic conversion. Data are presented as mean ± SD (*n* = 3, with technical repeats for each cattle). Asterisks indicate statistically significant differences (^*^P < 0.05; ^****^P < 0.0001; n.s., not significant). **E** Westen blot of myostatin and phosphorylated Smad3 protein from lysates of NC or *MSTN* KO skin fibroblasts (i). Relative band intensity against beta actin was compared. Data are presented as mean ± SD (ii) (*n* = 3). Asterisks indicate statistically significant differences (^**^P < 0.01; n.s., not significant)

**Fig. 3:**
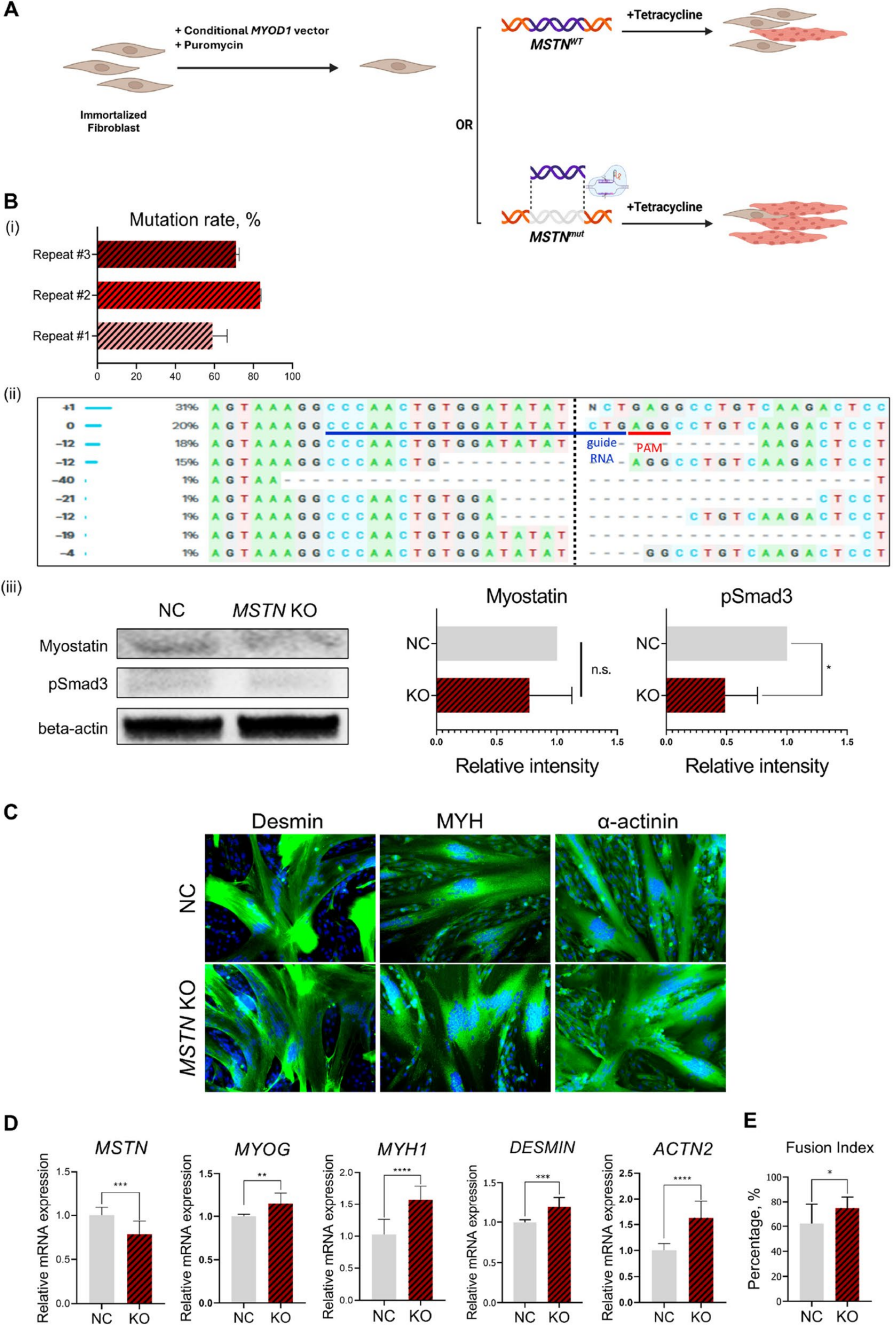
Effect of Myostatin treatment and *MSTN* KO in *MYOD1*-induced myogenic conversion of immortalized bovine fibroblast cell line. **A** Experimental scheme for Fig. 3B–E. The effect of *MSTN* KO in *MYOD1*-induced myogenic conversion was assessed. **B** Mutation rate (i) and representative mutation patterns at the guide RNA target site (ii) after inducing knockout in bovine immortalized fibroblast cell line, integrated with Doxycycline-dependent conditional MYOD1 expression vector. PAM = protospacer adjacent motif, Dotted line = predicted cut site by Cas9 protein. (iii) Westen blot of myostatin and phosphorylated Smad3 protein from lysates of NC or *MSTN* KO skin fibroblasts. Relative band intensity against beta actin was compared. Data are presented as mean ± SD (*n* = 3). Asterisks indicate statistically significant differences (^*^P < 0.05; ^***^P < 0.001; n.s., not significant). **C** Immunofluorescence images on d 7 after treatment of 2 μg/mL doxycycline, in WT cell or cells with *MSTN* KO. Green = immunofluorescent stain, Blue = Nuclei. Scale bar = 150 μm. **D** and **E** Semi-quantitative analysis of myogenesis factors (**D**) and fusion indexes (**E**) on d 7 after myogenic conversion. Data are presented as mean ± SD (*n* = 3). Asterisks indicate statistically significant differences (^*^P < 0.05; ^**^P < 0.01; ^***^P < 0.001; ^****^P < 0.0001)

**Fig. 4:**
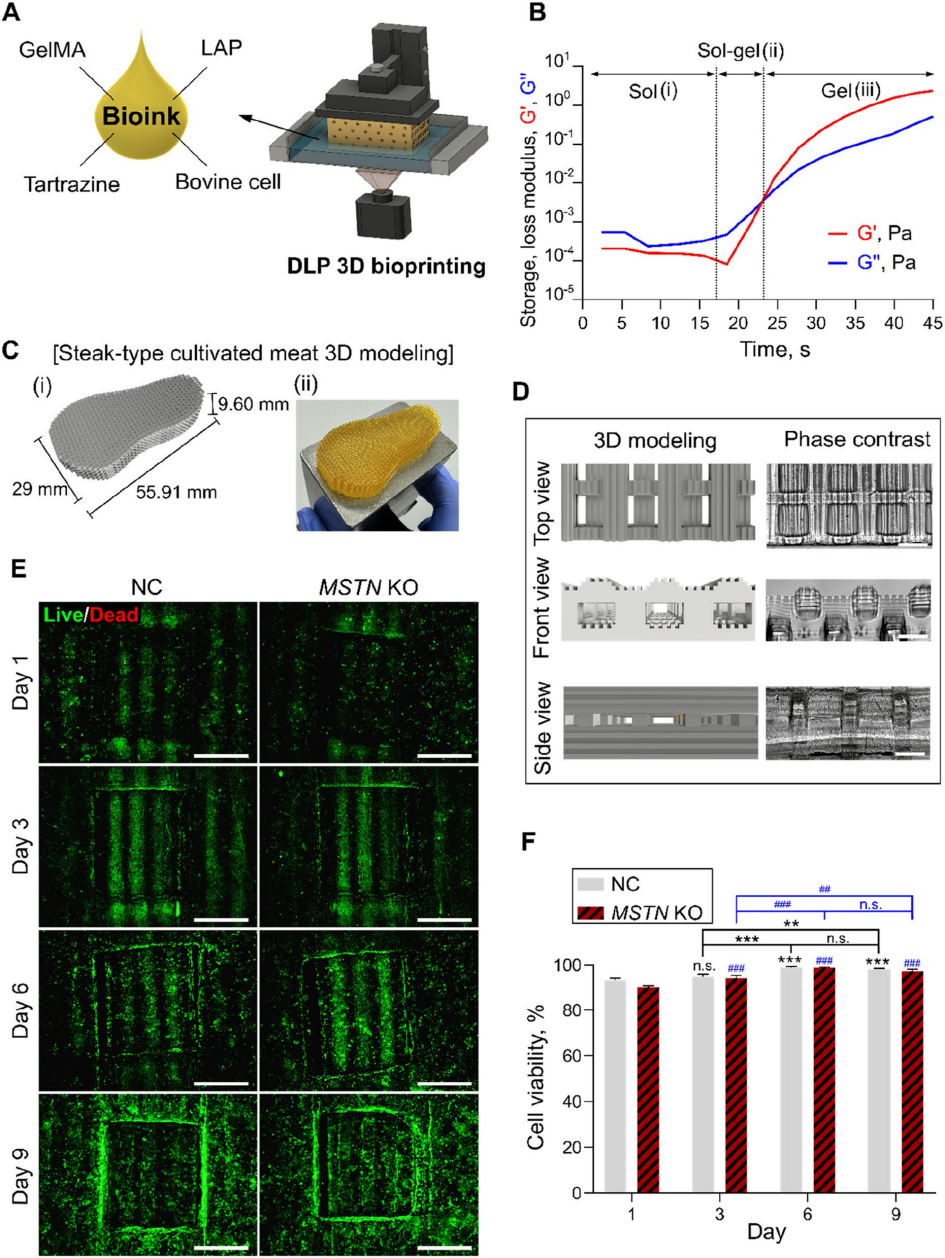
Schematic representation and analysis of the DLP bioprinting process, bioink composition, and cell viability in cultivated steak-type meat. **A** Schematic representation of the DLP bioprinting process for fabricating structures using bioink containing GelMA, LAP, tartrazine, and bovine cells. **B** Results of photorheological measurement of the bioink. **C** Schematic representation of 3D modeling of steak-type cultivated meat (i) and photograph (ii) (width, 29 mm, length, 55.91 mm, and height, 9.6 mm). **D** Schematic representation of the top, side, and front views of the enlarged portions of the 3D modeled structure and a phase-contrast image of the actual printed hydrogel. **E** Fluorescence microscopy images of NC and *MSTN* KO cells grown on GSH for 1, 3, 6, and 9 d in a Live/Dead assay. Green = Live, Blue = Dead. Scale bars = 500 μm. **F** Cell viability obtained by the Live/Dead assay. Data are presented as mean ± SD (*n* = 4). Asterisks indicate statistically significant differences (^**^P < 0.01; ^***^P < 0.001; n.s., not significant)

**Fig. 5:**
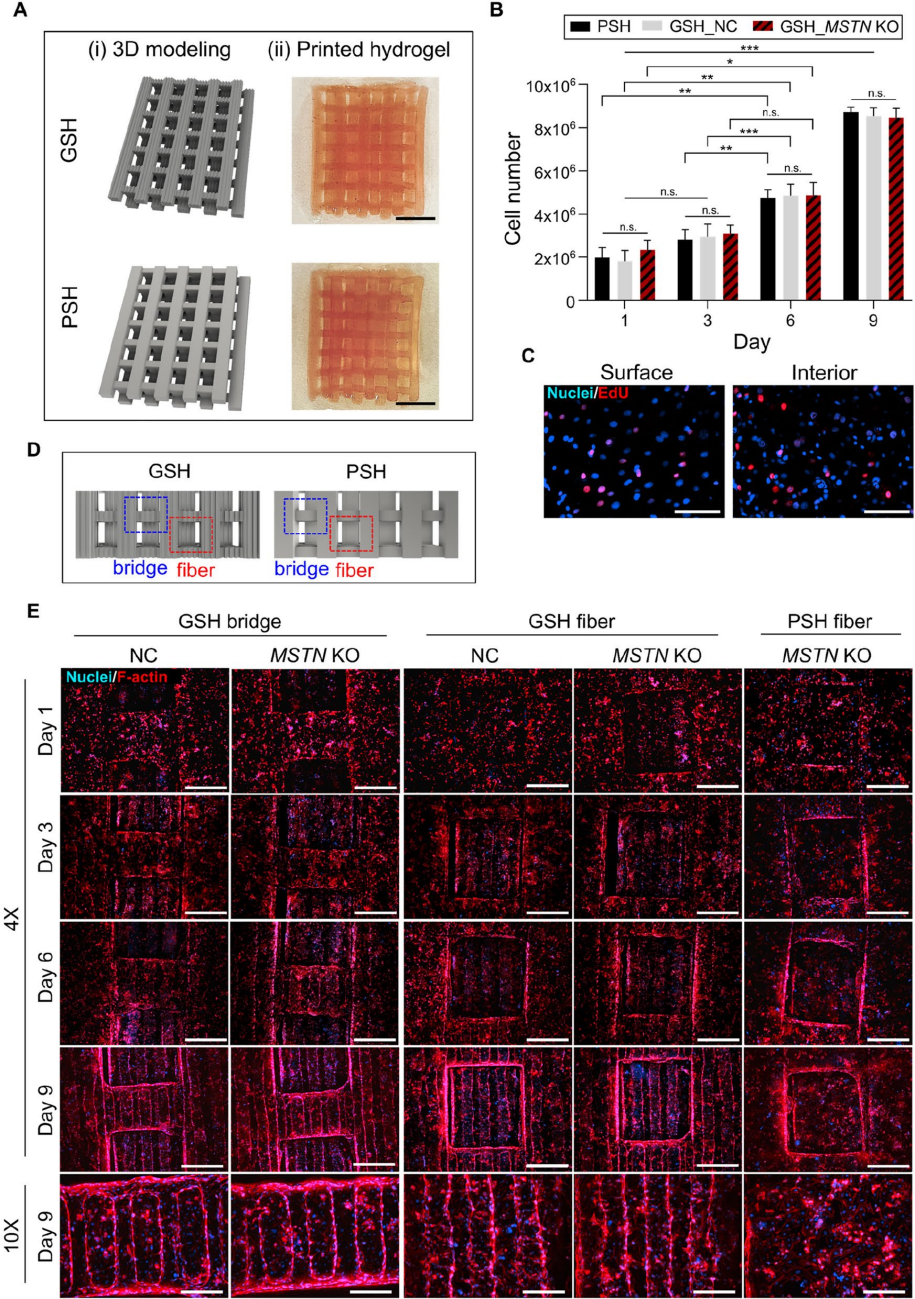
Proliferation of NC and *MSTN* KO cells in 3D-bioprinted GSH and PSH structures. **A** 3D modeling (i) and photograph (ii) of a portion of steak-type cultivated meat. Scale bars = 3 mm. **B** Cell counting for 1, 3, 6, and 9 d. Data are presented as mean ± SD (*n* = 4). Asterisks indicate statistically significant differences (^*^P < 0.05; ^**^P < 0.01; ^***^P < 0.001; n.s., not significant). **C** Fluorescence images from the EdU assay showing the proliferation of cells on the surface and interior regions of the hydrogel. Red = EdU, Blue = Nuclei. Scale bars = 100 μm. **D** Schematic representation of the fibers forming two intersecting layers (fiber, red) and the bridge (bridge, blue) connecting the fibers within the GSH and PSH hydrogels. **E** Fluorescence microscopy images of NC and *MSTN* KO cells grown on the GSH bridge, GSH fiber and PSH fiber for 1, 3, 6, and 9 d. Red = F-actin, Blue = Nuclei. Scale bars = 500 μm for 4X and 200 μm for 10X

**Fig. 6:**
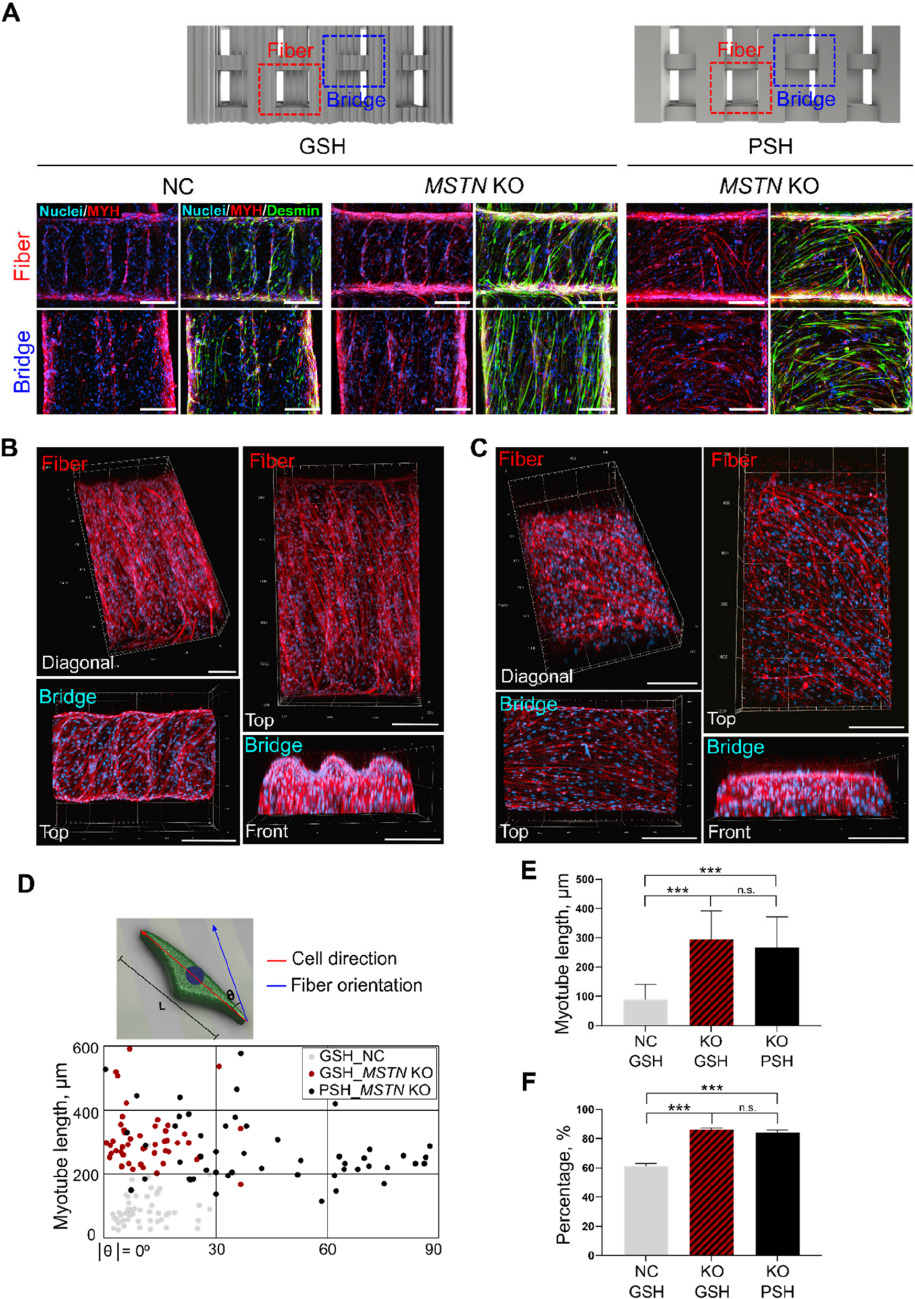
Myogenic differentiation and alignment of 3D-bioprinted NC and *MSTN* KO cells in GSH and PSH. **A** Immunofluorescence images of NC and *MSTN* KO cells grown on GSH and PSH, showing both fiber and bridge regions. Red = MYH, Green = Desmin, Blue = Nuclei. Scale bars = 200 μm. **B** 3D reconstruction images of *MSTN* KO myotubes in GSH hydrogels. Red = MYH, Blue = Nuclei. Scale bars = 200 μm. **C** 3D reconstruction of *MSTN* KO myotubes in PSH hydrogels. Red = MYH, Blue = Nuclei. Scale bars = 200 μm. **D** Schematic diagram representing the length of the myotube and the angle between the direction of the cell and the orientation of the fiber. The distribution of the angle and length of the myotube was obtained from immunofluorescence images of NC and *MSTN* KO cells grown on GSH and PSH. **E** Comparison of myotube lengths for NC and *MSTN* KO cells grown on GSH and PSH. Data are presented as mean ± SD (*n* = 5). Asterisks indicate statistically significant differences (^***^P < 0.001; n.s., not significant). **F** Fusion index of NC and *MSTN* KO cells grown on GSH and PSH. Data are presented as mean ± SD (*n* = 3). Asterisks indicate statistically significant differences (^***^P < 0.001; n.s., not significant)

**Fig. 7:**
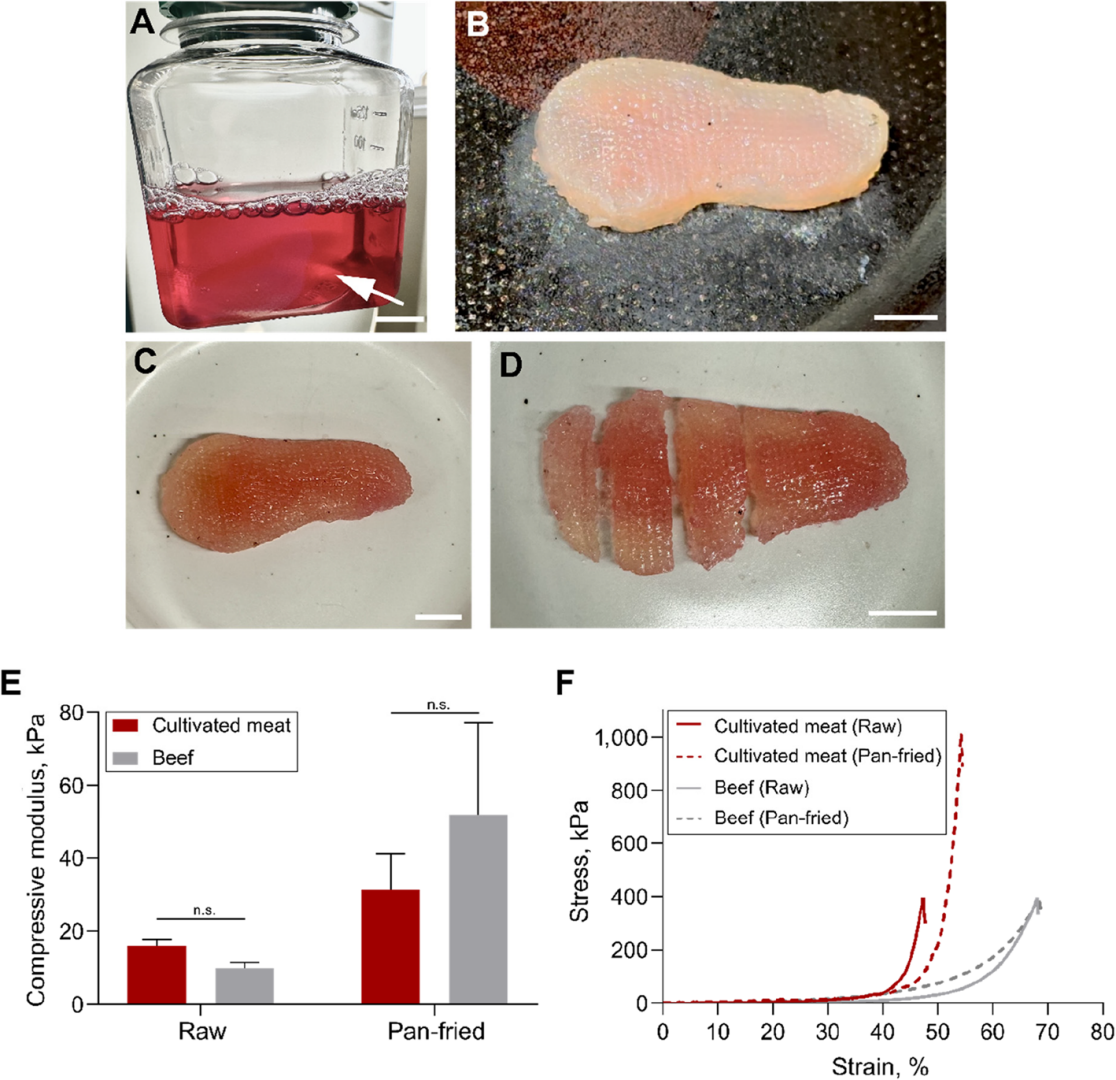
Preparation, cooking, and mechanical properties of 3D-bioprinted muscle-mimicking steak-type cultivated meat. **A** Photograph of steak-type cultivated meat after 3 weeks of culture in medium. **B** Photograph of steak-type cultivateed meat during pan-frying. **C** and **D** Photograph of cultivated steak-type pan-fried meat on dish (**C**) and sliced portions (**D**). Scale bars = 1 cm. **E** Compressive modulus of steak-type cultivated meat and beef (tenderloin) under two conditions: raw and pan-fried. Data are presented as mean ± SD (*n* = 6). Asterisks indicate statistically significant differences (n.s., not significant). **F** Representative stress–strain curves of steak-type cultivated meat and beef under two conditions: Raw and pan-fried

Data are presented as mean ± standard deviation (SD), and means were compared using an unpaired Student’s *t*-test, one-way analysis of variance (ANOVA) and two-way ANOVA followed by Tukey’s test, as appropriate. The mRNA level, fusion index, and relative band intensity in Figs. 1, 2 and 3 and Fig. S2 and S5 were compared between the two groups using Student’s *t*-test. The myotube length and fusion index in Fig. 6E and F were analysis using one-way ANOVA. The cell viability, cell number, and compressive modulus in Figs. 4F, 5B and 7E were analyzed using two-way ANOVA. Statistical significance was established at *P* < 0.05. All analyses were performed using GraphPad Prism v.8.0.2 (GraphPad Software, La Jolla, CA, USA).

## Results

### Non-viral overexpression of bovine *MYOD1* in primary bovine fibroblasts induced conversion into myocytes

To verify the conversion of primary dermal bovine fibroblasts into myocytes using a nonviral transfection method, bovine *MYOD1* coding sequences were cloned into a PiggyBac vector along with a ubiquitous promoter and green fluorescent protein (GFP) as a reporter (Fig. 1A). Expression vectors were transfected into primary bovine fibroblasts via electroporation. After 7 days of culture, GFP-positive cells produced multinucleated, desmin-positive cells, indicating that fibroblasts had converted to myocytes (Fig. 1B). However, these cells could not be passaged, making them unsuitable for large-scale culture (Fig. 1C). To overcome this issue, a doxycycline-dependent conditional *MYOD1* overexpression vector was designed to maintain fibroblast growth until a differentiation signal was generated (Fig. 1D). With the RFP reporter, only RFP-positive cells were sorted and differentiated (Fig. 1E and F). qPCR assays revealed that myogenesis-related factors increased with doxycycline treatment, and ICC showed that myogenic conversion was induced only by doxycycline (Fig. 1E and F). To sort cells transfected with myogenic vector, two selection methods using a red fluorescent protein (RFP) marker were tested: manually picking RFP + cells after single-cell culture and sorting them using flow cytometry. The number of multinucleated cells increased when sorted by flow cytometry, indicating that a reduced sorting time was beneficial for conversion, despite exposure to lasers (Fig. S1A and B). Additionally, no GFP signal was detected by flow cytometry, indicating that the conditional expression system operated without significant leakage (Fig. S1C). Subsequently, the fluorescent reporter was replaced with a puromycin resistance gene to facilitate ICC analysis using a fluorescent antibody (Fig. 1G). It was necessary to verify whether the newly constructed vector was functional. Considering the significant differences in transfection efficiency between primary cells and immortalized cell lines, a comparison was required between primary cells and the immortalized fibroblast cell line established in our previous study [17]. Consequently, using the vector containing the puromycin resistance gene, immunostaining confirmed the expression of myocyte-specific proteins, such as desmin, myosin heavy chain (MYH), and α-actinin, in both bovine primary cells (Fig. 1H and I) and immortalized cells (Fig. 1J and K). Furthermore, at the mRNA level, the expression of *MYOD1* and other myogenic markers increased upon doxycycline treatment.

### Fibroblasts from *MSTN* KO cattle efficiently and sensitively converted into myocytes via *MYOD1* using non-viral transfection

Skin fibroblasts from three WT or *MSTN* KO cattle, previously reported to have various mutation patterns and rates, were used for myogenic conversion. On average, when treated with 2 μg/mL of doxycycline, *MSTN* KO cells showed lower *MSTN* expression but higher *DESMIN* and *ACTN2* expression and a higher fusion index compared to NC cells from WT cattle (Fig. 2B–D). Interestingly, cells from KO cattle also showed greater conversion when treated with dilute concentrations of doxycycline (4 and 8 ng/mL) (Fig. S2). These results led us to hypothesize that *MSTN* KO would make *MYOD1*-mediated conversion more efficient. However, the results from primary fibroblasts were highly variable in both the NC and KO groups, requiring experiments in a controlled cell line to exclude variables from individual genetic backgrounds. Moreover, even the expression of phosphorylated Smad3 (pSmad3), a downstream protein directly involved in myostatin signaling, showed significant variability between individuals despite a significant decrease detected in myostatin protein expression, further emphasizing the need for controlled experiments using identical cells (Fig. 2E).

### Myostatin treatment decreased the myogenic conversion of bovine immortalized fibroblasts whereas *MSTN* KO increased it

Before myogenic conversion, immortalized fibroblasts were tested for cell stability and tumorigenicity. During ~ 100 doublings, the immortalized fibroblasts maintained stable viability, population doubling times, and morphologies (Fig. S3A and B). Also, no visible tumors were formed when injected to immunodeficient mice (Fig. S4A–C). A conditional *MYOD1* overexpression vector was introduced into the immortalized bovine fibroblasts by electroporation. Cells expressing a reporter were selected and used for further experiments. To investigate the effect of myostatin on *MYOD1*-induced myogenic conversion in bovine fibroblasts, cells were treated with medium containing mature myostatin (Fig. S5A–C). The presence of myostatin in the medium was confirmed by western blotting, and a subsequent increase in phosphorylated smad2/3 proteins was confirmed (Fig. S5D and E). No morphological changes or immunostaining signals were observed until d 4 of differentiation, with only weak signals detectable on d 7. Myostatin treatment also resulted in a decreased fusion index and lower expression of genes related to myogenesis. Subsequently, *MSTN* KO was induced in the same cell line (Fig. 3A). The average mutation rate was 71.11% ± 9.93%, with various mutation patterns (Fig. 3B (i) and (ii)). While the myostatin protein expression showed only a non-significant decreasing tendency, the pSmad3 protein expression significantly decreased with mutation induction (Fig. 3B (iii)). *MSTN* mRNA expression was reduced in the KO group compared with the NC control and consequently, myogenesis-related indices increased significantly with doxycycline treatment, consistent with the results of the myostatin treatment study (Fig. 3C–E). Finally, immortalized doxycycline-dependent convertible cells with or without *MSTN* KO were used for 3D bioprinting.

### Optimized fabrication and manufacturing of muscle-aligned steak-type cultivated meat with improved cell viability using DLP 3D bioprinting

In our previous study, we optimized the polymerization conditions for DLP bioprinting by adjusting variables such as printing speed, light intensity, exposure time, and wavelength using a yellow light absorber (tartrazine) to match the DLP light with the prepolymer [10]. This enabled the accurate fabrication of centimeter-sized steak-type cultivated meat with complex structures through the precise control of the microchannels.

For the effective development of cultivated meat, it is important not only to proliferate and differentiate muscle cells but also to mimic the structural arrangement of actual muscle bundles [45]. To achieve muscle alignment without additional post-processing after printing, we designed the 3D model of steak-type cultivated meat with 100-μm-scale fine groove structures to specifically guide muscle cell organization. Using a bioink composed of GelMA, LAP, tartrazine, and bovine cells, we applied the DLP bioprinting process (Fig. 4A). During photorheological measurements, the bioink transitioned to the hydrogel through a three-step process (sol, sol–gel, and gel steps). At 100-μm intervals, the bioink was exposed to LED light, initiating polymerization. Initially, the bioink remained in the sol state with a higher loss modulus (G'') than the storage modulus (G') due to its low viscosity with a light exposure time of 18.51 s (Fig. 4B (i)). Continued light exposure caused a sol–gel transition, marked by a rapid increase in the storage modulus (G') and a gelation time of 24.64 s, where G' intersected G'' (Fig. 4B (ii)). Further photo-crosslinking solidified the bioink into a gel (Fig. 4B (iii)). The optimal light exposure time for the formation of a 100-μm hydrogel was determined to be 24.64 s.

The overall structure of the cultivated meat was modeled as a centimeter-scale steak with a width of 29 mm, and was 55.91 mm in length and 9.6 mm in height (Fig. 4C (i)). A photograph of the actual printed steak-type cultivated meat shows an accurate reproduction of the 3D modeled structure through DLP bioprinting (Fig. 4C (ii)). Furthermore, we developed a GSH introducing 100-μm-sized groove structures throughout the hydrogel surface to further promote muscle cell alignment. Enlarged 3D modeling and phase-contrast photographs of the actual printed hydrogel reveal the microchannels and 100-μm-scale groove structures on the hydrogel surface from the top, side, and front (Fig. 4D). These grooves were specifically designed to promote the alignment and organization of muscle cells, mimicking the natural arrangement of muscle fibers.

The viability of NC and *MSTN* KO cells within DLP-printed GSH was evaluated over 1, 3, 6, and 9 d using a Live/Dead assay (Fig. 4E and F). High cell viability rates (> 95%) for both cell types up to 9 d after printing indicated that DLP printing with the GSH structure and GelMA bioink provided an appropriate environment for cell survival and proliferation.

### Proliferation of NC and *MSTN* KO cells encapsulated in GSH and PSH

We aimed to produce muscle-mimicking steak-type cultivated meat with a diameter of several centimeters, which presents a challenge for microscopic observation. Additionally, to examine cell behavior in GSH with 100-μm-scale groove structures and (plain-shaped hydrogel) PSH without groove structures, we produced partial slices of GSH and PSH composed of two intersecting layers throughout the structure of the cultivated meat (Fig. 5A (i)). A photograph of the printed hydrogel demonstrated that the 3D modeling was precisely replicated (Fig. 5A (ii)). This approach facilitates the enlargement of internal structures for cellular experiments and imaging, enabling the precise identification of microchannel and microgroove features.

To identify cell proliferation, 2 × 10^6^ NC and *MSTN* KO cells were encapsulated in bioink and 3D printed. Cells were cultured for 1, 3, 6, and 9 d post-printing, then retrieved after hydrogel digestion with collagenase and counted [46]. The cell number remained similar to the initial seeding density of 2.04 × 10^6^/mL on d 1 followed by gradual incremental increases to 8.57 × 10^6^/mL on d 9 (Fig. 5B). Structural differences between PSH and GSH and between NC and *MSTN* KO cells did not significantly affect proliferation rates. Cell proliferation within the hydrogel was also evaluated using an EdU assay, which enabled observation of cellular behavior in both the exterior and interior regions. On d 6 of culture, cells within the hydrogel were labeled with EdU and analyzed along the *z*-axis. The results demonstrated that cells were actively proliferating in both the surface and interior regions of the hydrogel (Fig. 5C and Additional file 2).

To examine cell behavior based on locations within the hydrogel, fibers that formed two intersecting layers (fiber, red) and fibers that were connected by a bridge (bridge, blue) within the GSH and PSH hydrogels were examined (Fig. 5D). To visualize cell proliferation, cells were fluorescently stained with phalloidin and DAPI and cell behavior was observed (Fig. 5E). On d 1, 3, 6, and 9 after culture, the bridge and fiber portions of GSH and the fiber portion of PSH were observed under magnification. Both NC and *MSTN* KO cells gradually increased in number and reached nearly 100% confluency by d 9. Furthermore, as the cells proliferated and arranged themselves along the groove structure in GSH, a distinct 100-μm-scale groove pattern was observed in both the bridge and the fiber regions on d 9. In PSH, although the cells showed significant proliferation, they did not exhibit any noticeable alignment or organized structures.

### Myogenic differentiation of 3D-bioprinted MSTN KO cells in GSH and PSH

To determine whether the microgroove structure facilitated muscle cell alignment, NC and *MSTN* KO cells were encapsulated in GSH and PSH, cultured for 9 d, and induced to differentiate with doxycycline. Myogenically differentiated cells within the hydrogels were visualized using fluorescence imaging after staining with MYH, desmin and DAPI (Fig. 6A). The fluorescence imaging demonstrated distinct myotube formation in both the fiber and bridge regions of the hydrogel. Notably, *MSTN* KO cells exhibited a significantly greater capacity for myogenic differentiation compared to NC cells, as evidenced by the increased number and extended length of the myotubes. In the GSH structure, *MSTN* KO cells formed densely packed and parallel-aligned myotube bundles that closely followed the microgroove patterns, suggesting enhanced structural organization and differentiation efficiency. In contrast, myotubes formed by NC cells were fewer in number, shorter in length, and exhibited less alignment along the microgroove structures.

To further investigate the alignment of myotubes in *MSTN* KO cells, 3D confocal microscopic imaging was performed. In GSH, dense and aligned myotubes following the groove structure were observed in the fiber and bridge regions (Fig. 6B). Conversely, in PSH, *MSTN* KO cells exhibited long myotubes that were randomly oriented and did not follow the direction of the fiber or bridge structures (Fig. 6C).

The degree of cell alignment was quantified by measuring the angle between the cells and the main axis of the fiber, as well as the length of the myotubes (Fig. 6D and E). In GSH, NC cells exhibited cell orientation angles between 2.3° and 28.4°, with relatively short myotubes averaging 90.38 ± 51.5 μm in length. In contrast, *MSTN* KO cells in GSH had cell orientation angles between 0.5° and 36.4° and significantly longer myotubes, averaging 303.7 ± 88.6 μm. In PSH, *MSTN* KO cells formed long myotubes with an average length of 273.2 ± 101 μm, but the cell orientation range was broader, ranging from 0.5° to 87.6°.

Additionally, *MSTN* KO cells exhibited a higher fusion index compared to NC cells in both GSH and PSH, regardless of the hydrogel structure (Fig. 6F). These findings indicate that the microgroove patterns in GSH do not impede myotube formation but instead promote dense and aligned myotube structures along the grooves, effectively mimicking natural muscle bundles.

### Preparation and cooking of 3D-bioprinted muscle-mimicking steak-type cultivated meat

A muscle-mimicking steak-type hydrogel construct (29 mm in width, 55.91 mm in length and 9.6 mm in height) was 3D bioprinted by encapsulating *MSTN* KO cells to function as representative samples. After culturing for 3 weeks, myogenic differentiation was induced using an established protocol (Fig. 7A). The cultivated meat was washed with PBS and pan-fried in a preheated cast iron pan (Fig. 7B). During cooking, the cultivated meat turned white, indicating cellular changes (Additional file 3). The pan-fried meat was removed from the pan and placed in a dish (Fig. 7C). The pan-fried meat retained its shape and could be sliced with a knife and fork, resembling real steaks (Fig. 7D and Additional file 4).

To evaluate the textural characteristics of cultivated meat in comparison to beef (tenderloin), the compressive modulus was measured (Fig. 7E). The results indicated that raw beef exhibited a relatively low modulus of 9.82 ± 1.43 kPa, while the cultivated meat with an aligned and stabilized structure demonstrated a higher modulus of 16.03 ± 1.65 kPa. Following pan-frying, the mechanical rigidity of beef increased significantly to 52.02 ± 1.43 kPa. A similar increase in rigidity was observed in the pan-fried cultivated meat, which achieved a modulus of 31.22 ± 9.09 kPa. Beef comprises various tissues, including muscle, fat, and connective tissue, which collectively contribute to its heterogeneous composition. This inherent heterogeneity introduced substantial variability during sample preparation using a biopsy punch, resulting in a large standard deviation in the compressive modulus results.

The strain–stress curve analysis revealed differences in elasticity between the cultivated meat and beef (Fig. 7F). Raw beef and pan-fried beef exhibited no significant differences in their strain–stress curves. In contrast, the yield point of the raw cultivated meat occurred at a strain rate of 47.25%, indicating the lowest elasticity among the groups. After pan-frying, the yield point of the cultivated meat increased to 54.5%, demonstrating an improvement in elasticity, though it remained lower than that of beef.

## Discussion

In this study, our objective was to develop efficient cell lines for meat production, using non-viral vectors. Among gene delivery methods, viral vectors have been widely used to produce *MYOD1*-mediated myogenic cell lines because of their higher efficiency [10, 11, 20]. However, viral vectors have disadvantages in the production and modification processes, particularly when researchers need to add or remove vector components. Non-viral vectors are generally known to have lower efficiency, but are considered a more flexible option due to their larger size capacity and ease of modification. In this study, we established an *MYOD1*-mediated myogenic cell line using a nonviral transposon vector. As shown in our previous studies, transposon-mediated gene delivery provides stable gene expression without causing health problems, even when integrated in vivo, making the cell lines produced in the current study more reliable [16, 47].

Genetic modification is another concern for food safety in cell-based meat production. Some groups claim that cisgenic genetic modifications, including *TERT*-induced immortalization, are relatively safe [48]. However, additional safety validation is required for cells with foreign genes inserted, regardless of the gene delivery method, as required by Food and Drug Administration regulations for any intentional genomic alteration [49–51]. As part of our safety validation of the *TERT*-induced immortalized cell lines, we confirmed that the immortalized fibroblasts used in the current study maintained their stability and lacked tumorigenicity (Fig. S3 and S4). This indicates that although telomerase is well known to be associated with tumors, *TERT* overexpression did not induce a pathogenic transformation in our cells. The Food and Drug Administration recently announced approval for *GGTA1*-edited hypoallergenic (GalSafe) pigs and issued a low-risk determination for *PRLR* gene-edited (SLICK) thermotolerance cattle [52, 53]. In Japan, *MSTN* KO sea breams are being produced and are currently available commercially [54, 55]. However, the cells developed in this study contain not only *TERT*-mediated immortalization but also the additional insertion of the *MYOD1* gene and a KO of the *MSTN* gene. As the safety of each genetic modification remains unassessed, thorough safety assessment would be required before make the cells created in this study could become commercially available, which is an unavoidable limitation of the current study.

Next, we attempted additional gene editing to make *MYOD1*-mediated myogenesis more efficient. The *MSTN* gene, which encodes myostatin and was first discovered in naturally occurring double-muscled cattle, was targeted for editing. Myostatin negatively regulates myogenesis and is a major target of gene editing to increase livestock productivity [5, 6, 26]. Therefore, we hypothesized that the myostatin protein or *MSTN* gene could also enhance *MYOD1*-mediated myogenesis. We found that treatment of cells with myostatin reduced myogenesis induced by exogenous overexpression of *MYOD1*, whereas removing the *MSTN* gene increased myogenesis-related indices, supporting our hypothesis.

Although bovine fibroblasts were successfully immortalized, and it was confirmed that conditional *MYOD1* overexpression combined with the CRISPR/Cas9 system improved the efficiency of *MYOD1*-induced myogenesis induction, several limitations related to the cell source need to be addressed in future studies. The first limitation is that the maturity of the induced myotubes was not observed beyond the cell fusion stage to a functional state. Under standard culture and differentiation conditions for naturally isolated myoblasts from mammalian species except rodents, contraction or striation are considered evidence for myotube functionality and this typically becomes evident after 11–14 d of differentiation, often requiring the addition of growth factors or the application of electrical pulses [56–58]. However, this study focused on evaluating the myogenesis efficiency of fibroblasts with *MSTN* KO. As a result, the differentiation period was limited to 7 d, sufficient for myotube formation, and the culture medium composition remained unchanged apart from the addition of doxycycline to activate the promoter. Consequently, while α-actinin and MYH were detected, a striated pattern was not observed, regardless of *MSTN* mutation status. The second limitation is that this study did not address the signaling pathway linking *MYOD1*-induced myogenesis and *MSTN* in detail. While this study demonstrated that myostatin suppresses myogenesis induced by *MYOD1* overexpression in a manner similar to its inhibition of natural myogenesis, where MSTN suppresses myogenesis by regulating MyoD expression levels, the previously proposed mechanism does not fully explain the observed results. Therefore, additional studies are needed to clarify the precise signaling pathways involved.

In terms of the 3D organization process, we successfully utilized *MSTN* KO cells in DLP 3D bioprinting platform to fabricate steak-type cultivated meat, allowing the production of muscle-mimicking constructs with precise structural characteristics. A bioink composed of GelMA hydrogel, LAP, tartrazine and bovine cells was prepared owing to its biocompatibility and the ability to form stable hydrogels upon photopolymerization [59, 60]. The printing conditions, including speed, light intensity, exposure time, and wavelength, were optimized based on our previous research to achieve high-resolution constructs [34]. The precisely controlled microchannels within the centimeter-sized steak-type cultivated meat facilitated the diffusion of nutrients to the internal regions, promoting cell survival and proliferation over extended culture periods. This characteristic is crucial for maintaining cell viability and ensuring uniform cell growth throughout large tissue constructs.

This study aimed to integrate 100-μm-scale groove patterns into hydrogel structures to enhance muscle cell proliferation and alignment. Previous studies demonstrated the effectiveness of groove patterns in facilitating muscle cell alignment [61, 62]. Based on these findings, we used 3D modeling and DLP bioprinting to precisely implement the 100-μm-scale groove pattern, resulting in the production of GSH with uniform application across the entire hydrogel surface. A comparative analysis of GSH and PSH revealed that both hydrogels supported similar levels of cell proliferation, thereby confirming that groove patterns did not impede cell growth. This finding establishes a foundation for the precise fabrication of centimeter-sized steak-type cultivated meat with muscle-aligned structures that closely resemble natural muscle fibers. After bioprinting, the cultivated meat constructs were further cultured to promote muscle formation. During this phase, the appropriate alignment of muscle fibers and the development of mature muscle tissue within the hydrogel matrix were successfully achieved. In particular, GSH demonstrated significantly higher efficacy in promoting muscle cell alignment than PSH. The 100-μm-scale groove patterns in GSH significantly enhanced muscle fiber alignment and organization relative to PSH. Furthermore, the use of *MSTN* KO cells resulted in improved muscle formation, producing muscle fibers that were both longer and more numerous than those derived from NC cells. Thus, high levels of muscle cell alignment can be achieved through the incorporation of groove patterns, eliminating the need for additional post-processing steps. These findings support the efficiency and practicality of our approach, which has the potential to significantly enhance the productivity of cultivated meat production and presents a viable strategy for large-scale applications. Therefore, this study not only advances the field of cultivated meat production, but also provides a robust foundation for scaling up to industrial levels. Also, the results in Fig. 7 indicate that while the texture and elasticity of cultivated meat have not yet achieved parity with actual beef, the cooking process significantly enhances its mechanical properties. The observed improvements in rigidity and elasticity highlight the potential of cultivated meat to approximate the textural characteristics of beef. Further optimization of cell proliferation and differentiation, as well as structural refinement, is expected to enhance the mechanical and textural properties of cultivated meat, thereby facilitating its commercial viability.

However, several limitations of this study must be addressed. The primary challenge is to identify a suitable non-animal-based bio-ink to replace the GelMA hydrogel, which is currently derived from animal sources. For instance, SerMA (i.e., sericin methacryloyl), a bioink based on sericin extracted from silk, is a biocompatible and photopolymerizable material that represents a potential alternative to animal-based bioinks [63, 64]. One of the challenges of the hydrogel system fabricated through DLP printing is the presence of a layered wall structure with a specific thickness, determined by the layer-by-layer printing process [65]. This structure can interfere with the proliferation of cells inside the gel and restrict the diffusion of essential substances such as nutrients and oxygen. To address this challenge, future research should prioritize strategies to enhance the internal space available for cell growth. This can be achieved through the incorporation of a large number of precisely engineered microchannels within the gel [10, 65], or by employing sacrificial hydrogels to regulate the size and distribution of internal pores [66, 67]. Such advancements are expected to substantially improve cell viability and facilitate efficient nutrient exchange within the hydrogel system, thereby overcoming the inherent limitations of DLP 3D printed hydrogels in applications related to cultivated meat production.

Additionally, the stability and biocompatibility of LAP, a photoinitiator used in bioinks, must be rigorously evaluated to ensure its long-term safety and functionality in applications related to cultivated meat. Variability in cell proliferation and differentiation rates is influenced by numerous factors, including the cell source and the specific conditions of bioprinting and culturing processes. Ensuring the consistency and reproducibility of these parameters is critical for the successful commercialization of cultivated meat products. Also, the doxycycline-mediated expression system could be replaced with other non-toxic conditional expression systems such as a cumate-mediated system [68].

## Conclusions

In this study, cell genetic modification and 3D DLP bioprinting with groove-patterned hydrogels were combined to produce high-quality muscle-aligned cultivated meat. The proposed method can significantly improve the efficiency and scalability of meat production. Despite the aforementioned challenges, this study presents several notable advantages. Achieving myogenic differentiation using *MSTN* KO cells and groove-patterned hydrogels is a promising approach for cultivated meat production. This method simplifies the production process by eliminating the need for additional alignment steps, thereby enhancing efficiency and scalability. Furthermore, the successful integration of *MSTN* KO cells enhances myogenic differentiation and results in structurally aligned muscle tissues. Despite the limitation that the results were obtained from non-myogenic cells modified to be myogenic, the current findings that *MSTN* KO cattle cells undergo more efficient myogenic conversion than NC cells suggest implications for future research directions using myoblasts (Fig. 2C–E and Fig. 6). Although various groups have generated *MSTN* KO cattle with increased muscle phenotypes, this report is the first to verify that *MSTN* KO cattle cells are more efficient for cultivated meat production. This highlights the significance of genetic modifications in donor animals or cells in cultivated meat production [6, 25–27, 69]. Consequently, this study not only advances the field of cultivated meat production but also provides a robust foundation for scaling up to industrial levels.

## Supplementary Information

Additional file 1. Fig. S1. Effect of strategies for selecting RFP+ cells on the formation of multinuclear cells. Fig. S2. The difference in sensitivity to *MYOD1*-induced myogenesis between wildtype and MSTN KO cells. Fig. S3. Proliferation of bovine *TERT*-induced immortalized fibroblasts. Fig. S4. In vivo tumorigenicity assay of bovine *TERT*-induced immortalized fibroblasts.

Additional file 2. Supplementary Video 1 Z-stack imaging of EdU stained cell encapsulated by hydrogel.

Additional file 3. Supplementary Video 2 Pan-frying cultivated meat.

Additional file 4. Supplementary Video 3 Slicing pan-fried cultivated meat with a knife and fork.

## Data Availability

The datasets generated and/or analyzed during this study are available from the corresponding author on reasonable request.

## Abbreviations

DLP: Digital light processing
GelMA: Gelatin methacryloyl
GFP: Green fluorescent protein
GSH: Groove-shaped hydrogel
ICC: Immunocytochemistry
KO: Knockout
MA: Methacrylic anhydride
NC: Negative control
PSH: Plain-shaped hydrogel
RNP: Cas9-gRNA complex
TERT: Telomerase reverse transcriptase
WT: Wildtype
