# Supplementary material for: MSTN knockout enhances the production of MYOD1-mediated steak-type cultivated meat
Source: J Anim Sci Biotechnol. 2025 Mar 11;16:41. doi: 10.1186/s40104-025-01173-1 (PMC11895244; doi:10.1186/s40104-025-01173-1)
Supplement: Supplementary file 1 — Additional file 1. Fig. S1. Effect of strategies for selecting RFP+ cells on the formation of multinuclear cells. Fig. S2. The difference in sensitivity to MYOD1-induced myogenesis between wildtype and MSTN KO cells. Fig. S3. Proliferation of bovine TERT-induced immortalized fibroblasts. Fig. S4. In vivo tumorigenicity assay of bovine TERT-induced immortalized fibroblasts. [file 40104_2025_1173_MOESM1_ESM.docx]

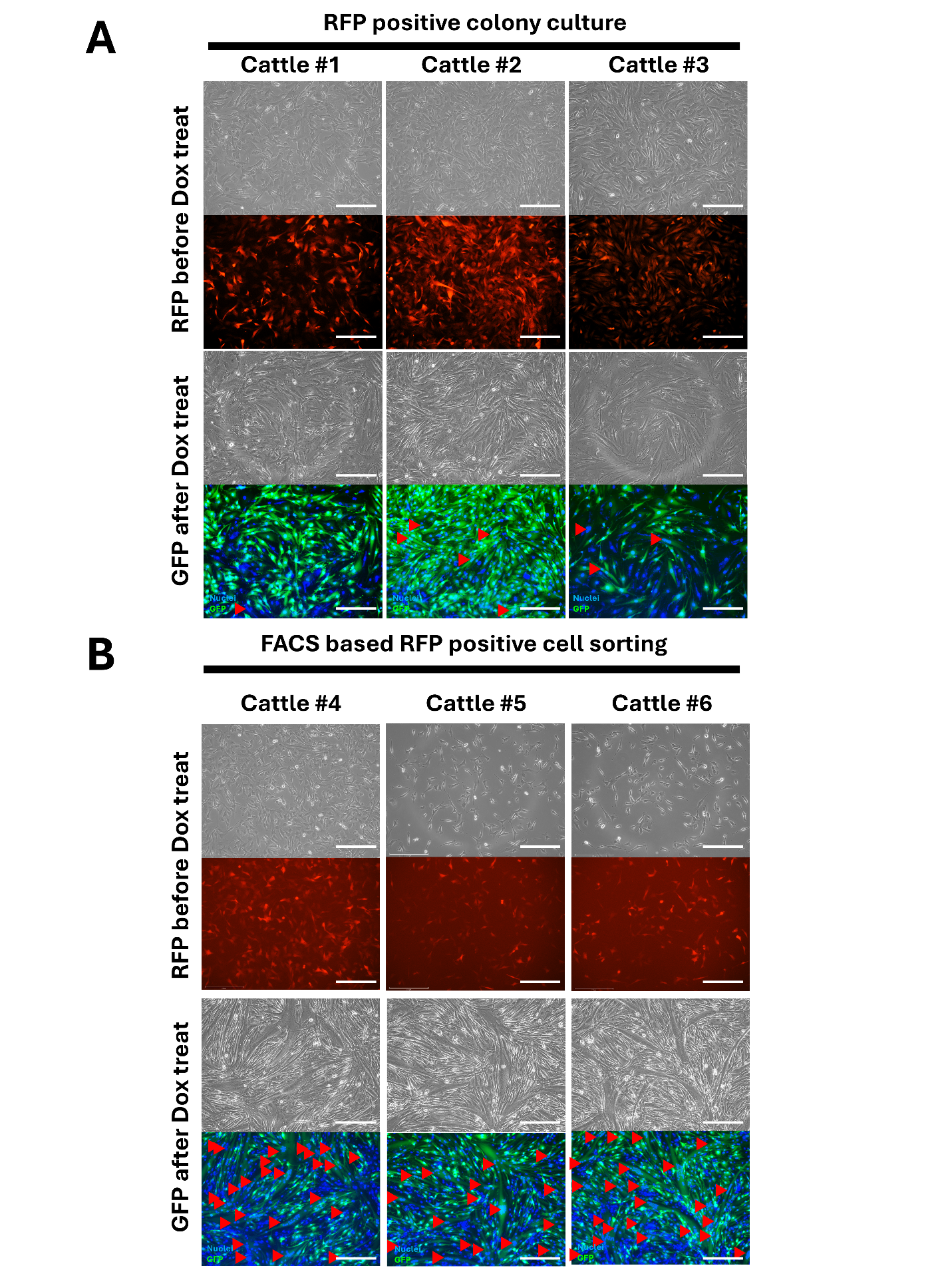

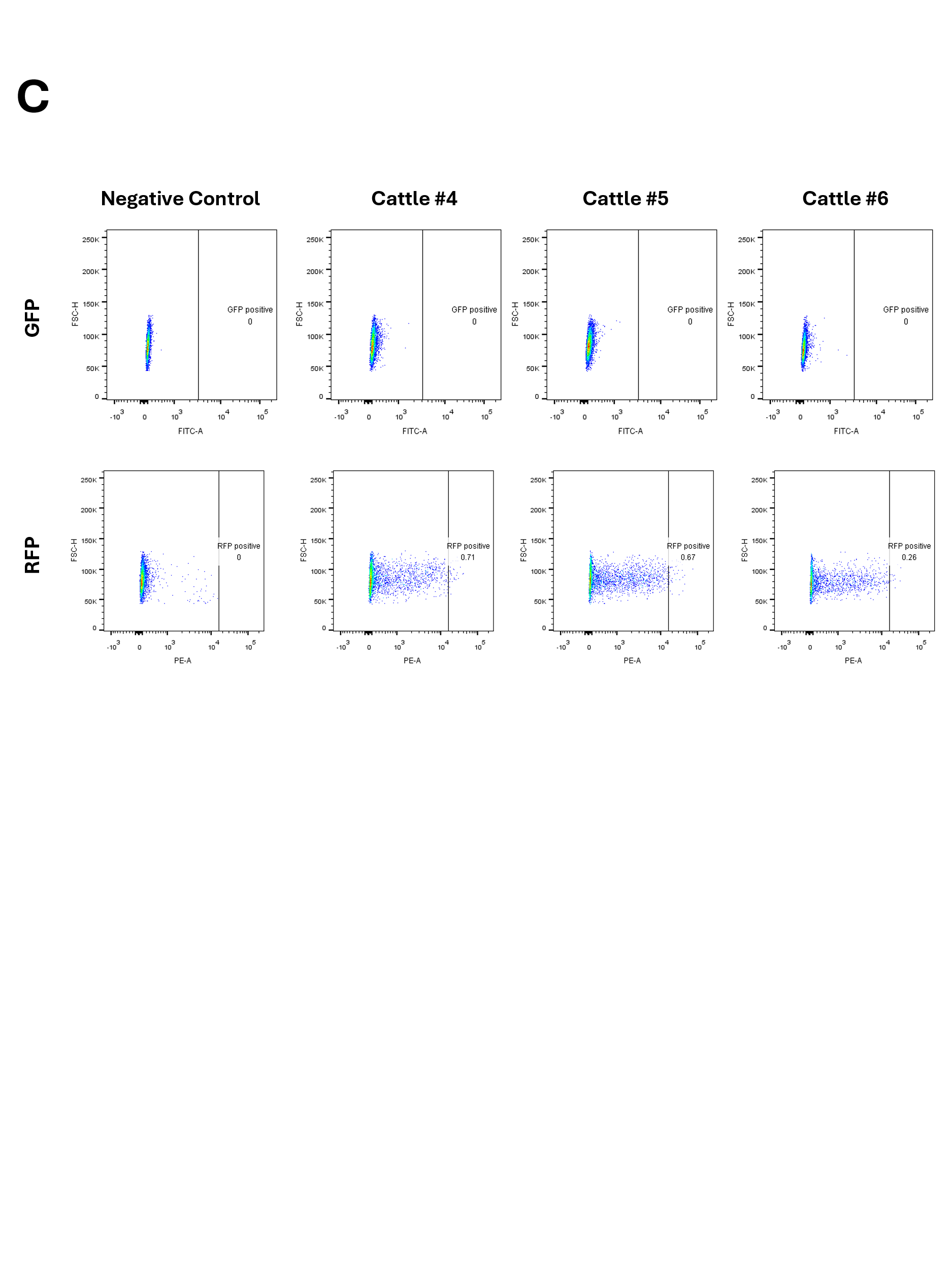


**Figure S1. Effect of strategies for selecting RFP+ cells on the formation of multinuclear cells.** (A) Bright and fluorescent images on before or day 7 after doxycycline treatment. Cells induced with vectors were picked manually. Red arrowhead = multi-nucleated cells, Scale bar = 275 μm. (B) Bright and fluorescent images on before or day 7 after doxycycline treatment. Cells with red fluorescent reporter signal were classified using flow cytometry. Red arrowhead = multi-nucleated cells, Scale bar = 275 μm. (C) Results of flow cytometry. Cells with excitation signal at 488 nm wavelength (a, b, and c) and 594 nm (a’, b’, and c’). P5 = cells with positive signal.


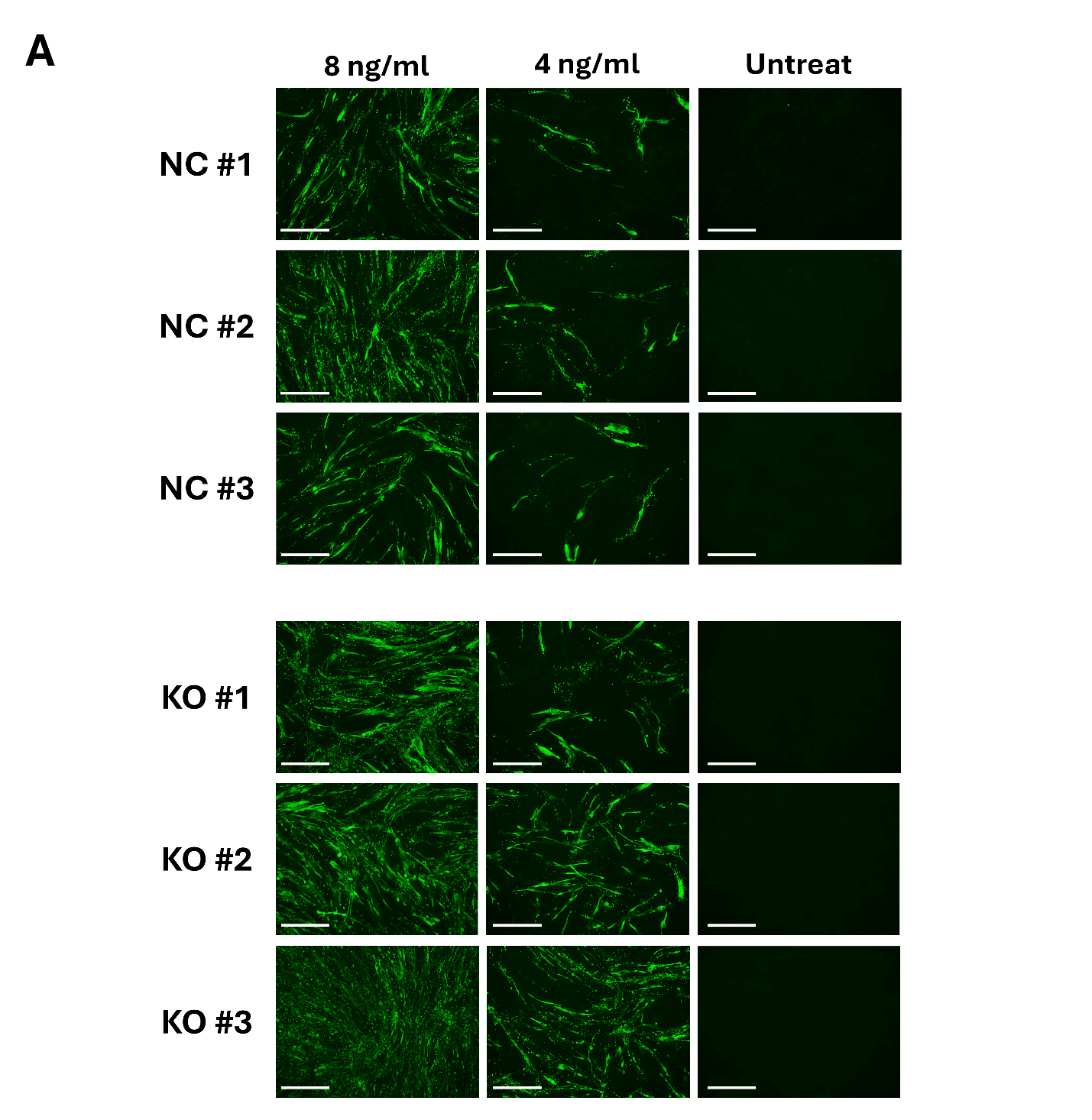


**Figure S2**. **The difference in sensitivity to *MYOD1*-induced myogenesis between wildtype and *MSTN* KO cells.** (A) ICC using anti-desmin antibody after myogenic conversion of NC or *MSTN* KO skin fibroblasts with diluted doxycycline. 4 and 8 ng/ml of doxycycline was treated for 7 days. NC = negative control. Green = Desmin. Scale bar = 300 μm.


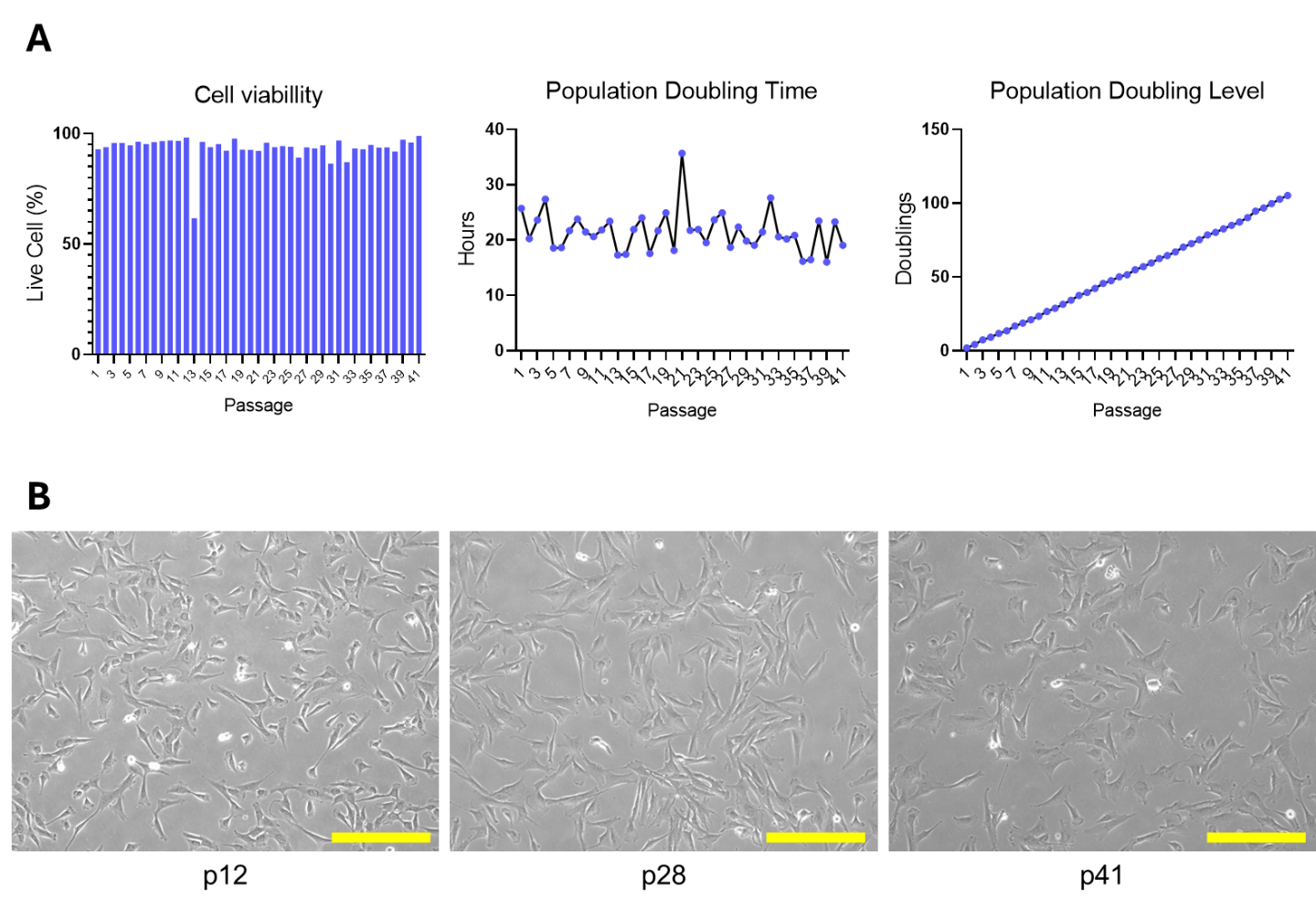


**Figure S3. Proliferation of bovine TERT-induced immortalized fibroblasts.** (A) Cell viability and population doublings during long term culture of the immortalized bovine fibroblast used in current study. (B) Representative images of immortalized fibroblast morphology during immortalization check. Scale bar = 300 μm.


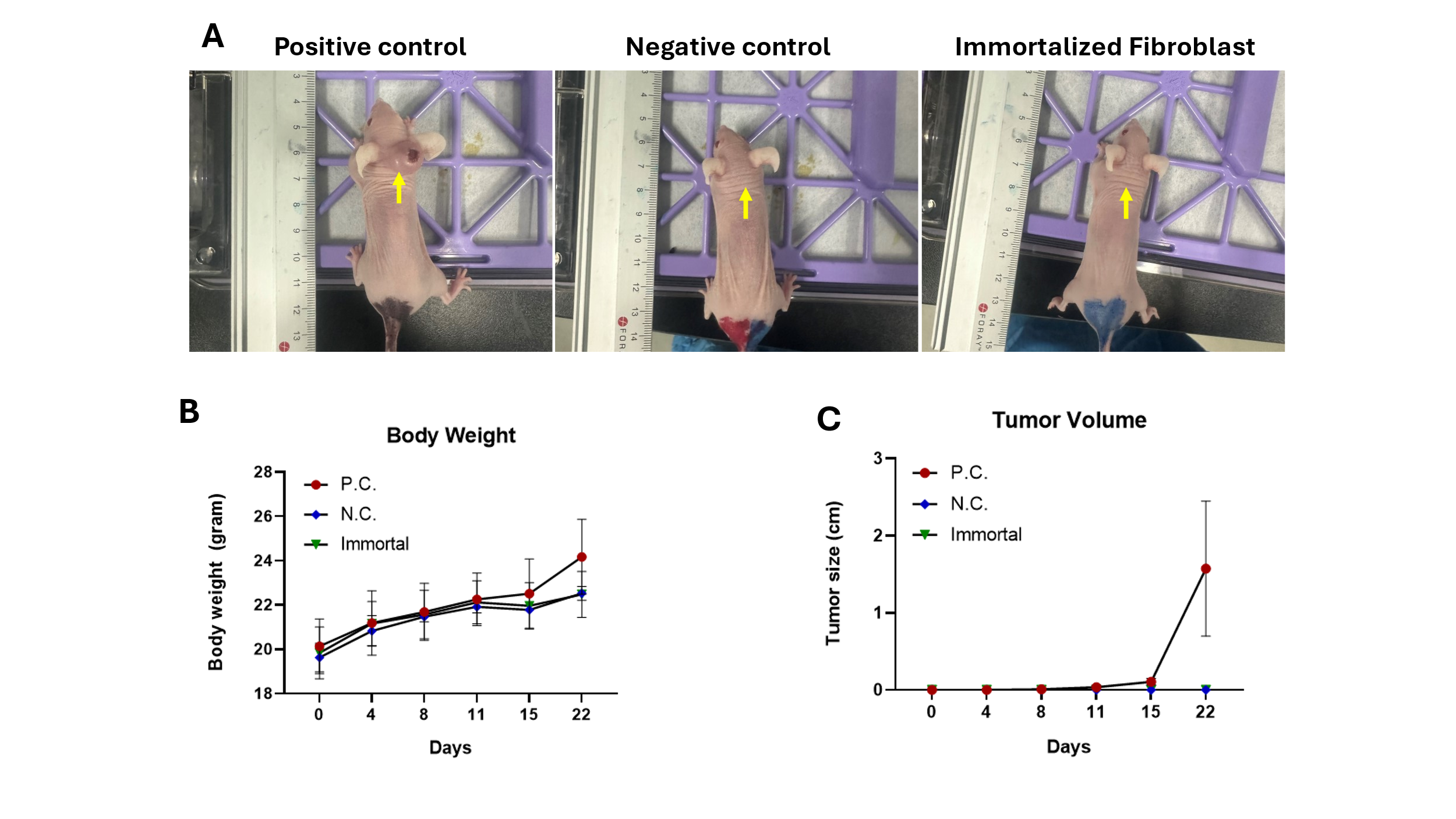
 **Figure S4. *In vivo* tumorigenicity assay of bovine *TERT*-induced immortalized fibroblasts.** (A) Representative images of the tumor formation test in immunodeficient mice. The 4T1 breast cancer cell line was used as a positive control and the primary fibroblast was used as a negative control. (B) Trend in body weight of mice injected with each cell. P.C. = positive control, N.C. = negative control. (C) Size of tumor formed at the injection site. P.C. = positive control, N.C. = negative control.

*
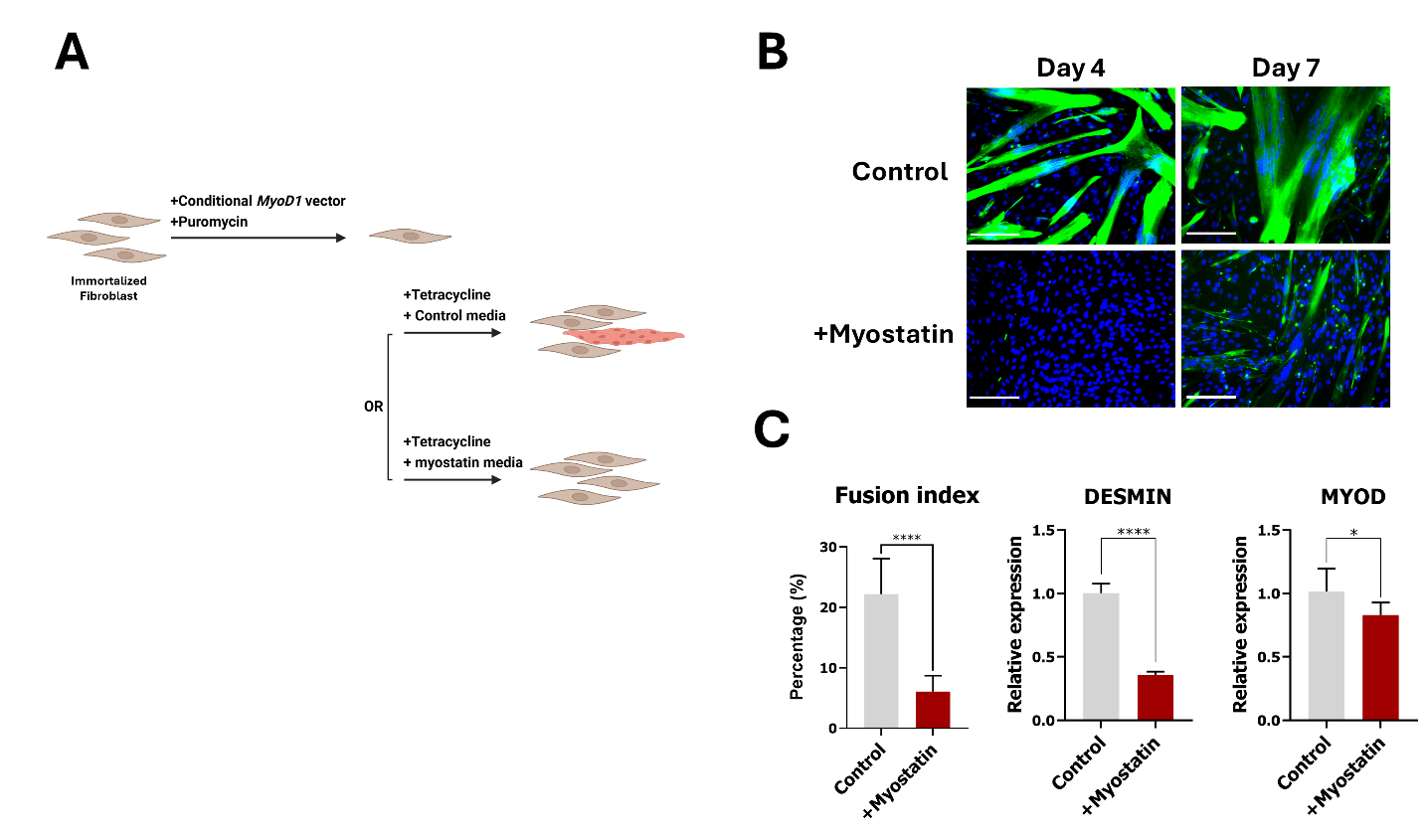

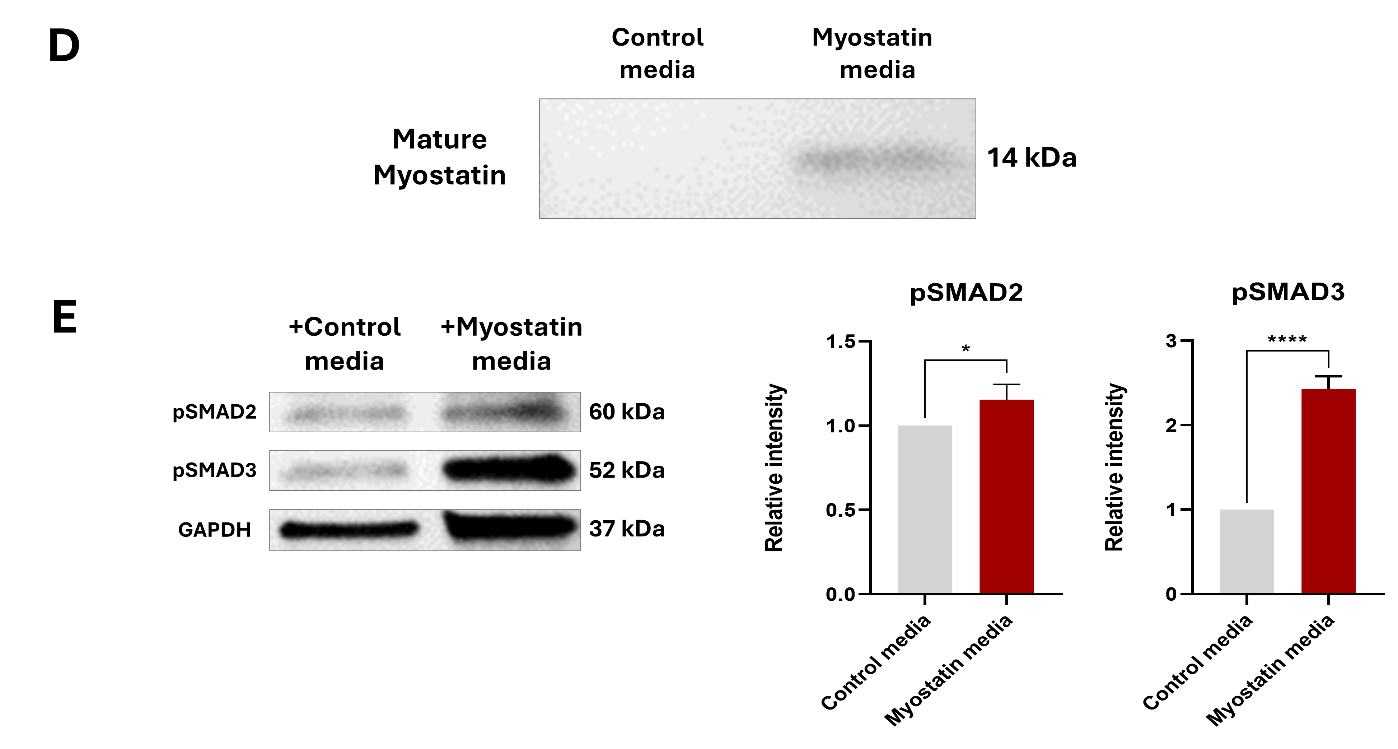
*

**Figure S5.** (A) Schematic representation of experiments. Effect of myostatin treatment in *MYOD1*-induced myogenic conversion was assessed. (B) Immunofluorescence images using anti-desmin antibody 4 days or 7 days after treatment of 2 μg/ml doxycycline, in control media or myostatin-containing media. Green = Desmin, Blue = Nuclei. Scale bar = 150 μm. (C) Semi-quantitative analysis of myogenesis related factors and fusion index on day 7 after myogenic conversion in each media. Data are presented as means ± SD (n = 3). Asterisks indicate statistically significant differences (*p < 0.05; ****p < 0.0001). (D) Western blotting of the media used in supplementary figure 4A-4C, using anti-myostatin antibody. (E) Western blotting assay for the detection of phosphorylated Smad2/3 protein in cells with *MYOD1*-induced myogenic conversion in myostatin-conditioned media treatment. The relative band intensity of phosphorylated Smad2/3 was compared with the band of the homeobox gene. Fold changes in relative intensity between two groups were calculated. Asterisks indicate statistically significant differences. * *P*  < 0.05, ** *P* < 0.01, *** *P* < 0.001, **** *P* < 0.0001.
